# Twist Family BHLH Transcription Factor 1 orchestrates hypoxia-lactate crosstalk to bridge glycolytic flux and tubular maladaptation in fibrotic kidneys

**DOI:** 10.1186/s43556-026-00555-9

**Published:** 2026-08-24

**Authors:** Zhixiang Yu, Yuzhan Zhang, Xiao Bai, Jin Zhao, Lei Wei, Jingli Gao, Hao Wu, Rui Ma, Xiaoxuan Ning, Shiren Sun

**Affiliations:** 1 Department of Nephrology, Xijing Hospital, Fourth Military Medical University, Xi’an, China; 2 Department of Geriatrics, Xijing Hospital, Fourth Military Medical University, Xi’an, China; 3 Department of Nephrology, The Ninth Medical Center of Chinese PLA General Hospital, Beijing, China

**Keywords:** AKI-CKD, Renal tubular epithelial cells, Maladaptive repair process, Twist1, Lactylation, Glycolysis

## Abstract

The reprogramming of glycolytic metabolism is essential for maintaining the reparative homeostasis of tubular epithelial cells (TECs). Maladaptive TEC repair mechanisms drive the progression from acute kidney injury (AKI) to chronic kidney disease (CKD). Twist Family BHLH Transcription Factor 1 (Twist1) has been implicated in epithelial-mesenchymal transition and metabolic dysregulation in cancer and fibrotic diseases. Twist1 promotes renal fibrosis through lipid accumulation in TECs by transcriptionally regulating genes involved in lipid metabolism. The role of Twist1 in glycolytic reprogramming during the transition from AKI to CKD was investigated. Tubular Twist1 knockout mice, RNA sequencing, metabolomics, and in vivo and in vitro experiments were used to evaluate the function and mechanism of Twist1-regulated glycolytic reprogramming. Twist1 orchestrated a dual metabolic insult of fatty acid oxidation suppression and glycolysis hyper-activation, thereby rewiring TEC processes toward a pro-fibrotic phenotype. Hypoxia-induced Twist1 activated hexokinase-2-driven glycolysis and amplified lactate accumulation, thereby stabilizing Twist1 expression through epigenetic modifications. Multidimensional studies demonstrated that lactate significantly enhanced maladaptive repair processes in TECs and contributed to renal fibrosis through a positive feedback loop that exacerbated glycolysis. Mechanistically, lactylation of histone H3 at K18 (H3K18lac) upregulated Twist1 transcription, which in turn accelerated hexokinase-2 expression and further promoted glycolytic activity. This study elucidates a lactate/H3K18lac/Twist1/hexokinase-2 axis as a central mechanism driving AKI-to-CKD progression. Twist1 emerges as a hypoxia-responsive transcription factor that amplifies glycolysis in a self-reinforcing cycle that fuels fibrosis. This study uniquely positions Twist1 as a hypoxia-responsive transcription factor in renal fibrosis and implicates lactate in autocrine TEC reprogramming.

**Supplementary Information:**

The online version contains supplementary material available at 10.1186/s43556-026-00555-9.

## Introduction

Acute kidney injury (AKI) not only causes high short-term mortality [1] but also progresses to chronic kidney disease (CKD) in 20%–50% of survivors [2]. CKD resulting from AKI remains an unresolved clinical challenge because of difficulties in prevention and limited therapeutic options [3, 4]. During AKI, renal tubular epithelial cells (TECs), key initiators of injury and fibrosis [5], undergo maladaptive repair characterized by persistent dedifferentiation and metabolic reprogramming [6]. Although a transient shift from fatty acid oxidation (FAO) to glycolysis is adaptive after AKI [7], sustained glycolytic activation in TECs promotes maladaptive repair and renal fibrosis [8, 9]. However, the mechanisms underlying persistent glycolysis remain poorly understood.

Twist Family BHLH Transcription Factor 1 (Twist1), a basic helix–loop–helix transcription factor, has been implicated in epithelial–mesenchymal transition and metabolic dysregulation in cancer and fibrotic diseases. In the kidney, previous work demonstrated that Twist1 promotes renal fibrosis through lipid accumulation in TECs by transcriptionally regulating genes involved in lipid metabolism [6]. However, its role in glycolytic reprogramming during the AKI-to-CKD transition remains unexplored. Previous findings further demonstrated that hypoxia induces the initial upregulation of Twist1 in TECs, in which hypoxia-inducible factor 1α (HIF1α) directly binds to a functional hypoxia-responsive element in the proximal *Twist1*promoter, thereby driving its transcriptional activation [10].

Lactate, the terminal product of glycolysis, is increasingly recognized not as a waste metabolite but as a signaling molecule and substrate for histone lactylation, a post-translational modification implicated in gene regulation [11–13]. In sepsis-induced AKI, lactate overproduction promotes renal injury through lactylation of specific effector proteins, and recent reports have highlighted the contribution of histone lactylation to the AKI-to-CKD transition [14]. However, whether a lactate–histone lactylation axis operates in TECs to sustain glycolytic gene expression and maladaptive repair during progressive renal fibrosis remains unexplored. Defining this epigenetic circuit could fundamentally revise the understanding of how metabolic byproducts perpetuate structural kidney damage.

This study hypothesized that hypoxia-induced Twist1 transcriptionally activates hexokinase-2 (HK2)-driven glycolysis in TECs, leading to lactate accumulation that, through histone H3K18 lactylation (H3K18lac), feeds back to stabilize Twist1 expression. This self-reinforcing loop may sustain glycolysis and promote maladaptive repair and fibrosis. To test this hypothesis, proximal tubule (PT)-specific *Twist1* knockout mice, transcriptomic and metabolomic profiling, and functional studies in murine AKI-to-CKD models and hypoxic HK-2 cells were employed. The results delineate a lactate/H3K18lac/Twist1/HK2 axis as a central mechanism linking metabolic dysregulation to tubular maladaptation. This study identifies Twist1 as a hypoxia-responsive transcription factor that amplifies glycolysis through an epigenetic feedback circuit and uniquely implicates lactate in the autocrine reprogramming of TECs, providing a mechanistic rationale for targeting this axis to interrupt AKI-to-CKD progression.

## Results

### Twist1 orchestrates metabolic reprogramming to drive tubular injury-fibrosis transition

**Fig. 1:**
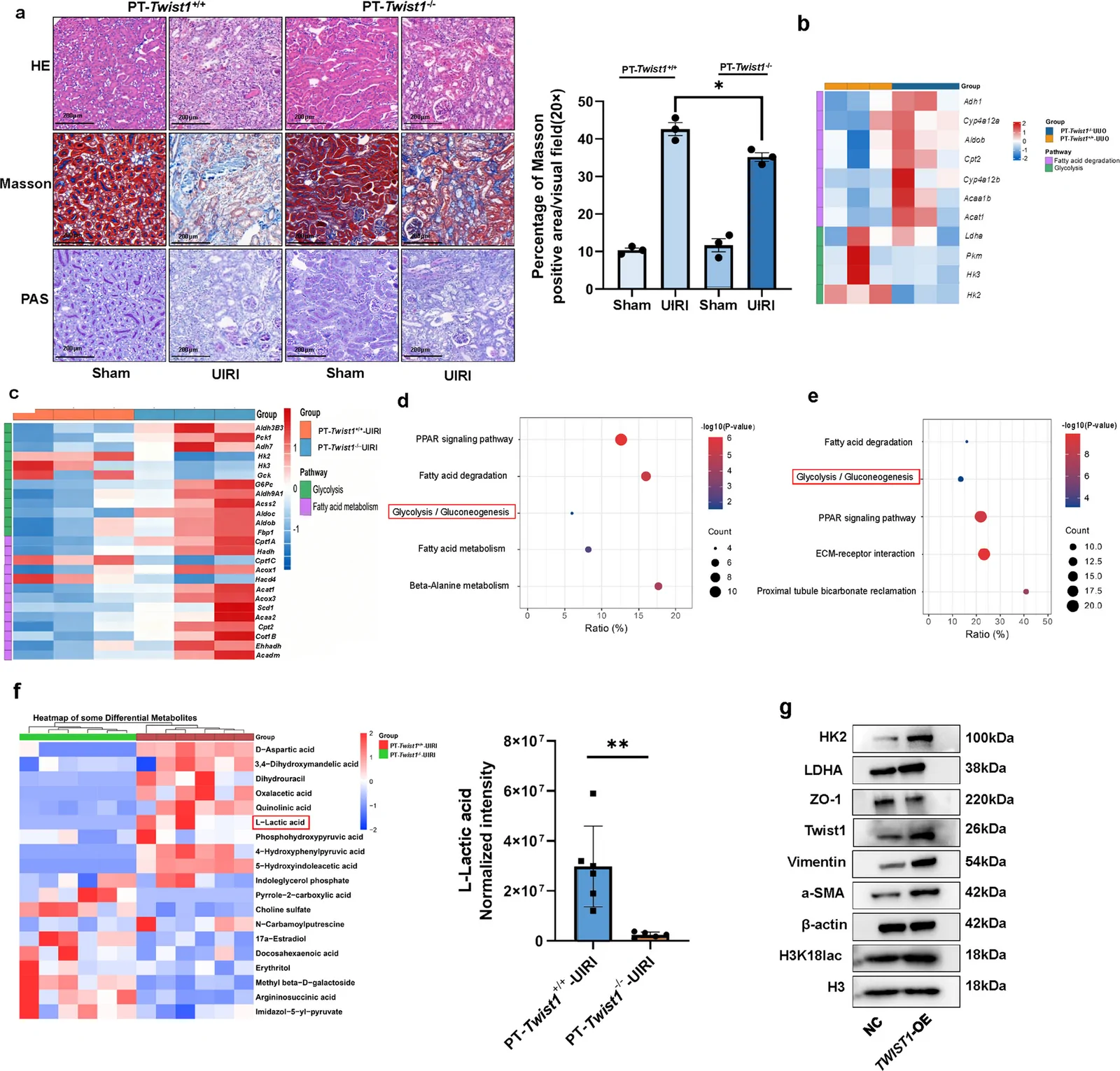
Twist1 deficiency alters glycolysis, fatty acid metabolism, and tubular dedifferentiation in kidney injury models. **a** HE, Masson, and PAS staining methods were used to detect the morphology and degree of fibrosis in the kidneys of sham mice and Day 14 UIRI mice respectively. The magnification was 20 ×. Statistical analysis of collagen area, comparing the *Twist1* knockout (PT-*Twist1*^–/–^) UIRI group with the (PT-*Twist1*^+/+^) group, *P < 0.05, n = 3 mice per group. **b** Heat map of differential genes related to glycolysis and fatty acid metabolism pathways in kidney tissue of PT-*Twist1*^+/+^ (sham) mice and PT-*Twist1*^–/–^ UUO mice (from GSE60685), and in **c** PT-*Twist1*^+/+^ (sham) mice and PT-*Twist1*^–/–^ UIRI mice. *P < 0.05, n = 3 mice per group. **d** The enrichment diagram of differential gene pathways in kidney tissue of PT-*Twist1*^+/+^ (sham) mice and PT-*Twist1*^–/–^ UUO mice, and in **e** PT-*Twist1*^+/+^ (sham) mice and PT-*Twist1*^–/–^ UIRI mice. The size and color of bubbles represent the number of genes enriched in the pathway and the P value of the enrichment. **f** Heatmap of a part of differences in untargeted metabolomics intermediates in whole kidney tissue of PT-*Twist1*^–/–^ and PT-*Twist1*^+/+^ UIRI mice. Lactate abundance in the kidney tissue of two groups of mice, **P < 0.01, n = 6 mice per group.** g** Western blotting detects the dedifferentiation markers and the expression levels of key glycolysis enzymes in HK-2 cells following *TWIST1* overexpression (OE)

To delineate the functional role of Twist1 in renal pathophysiology, marked Twist1 upregulation in TECs from patients with CKD was first demonstrated by immunohistochemical analysis of human kidney biopsies (Fig. S1a). This finding prompted a systematic investigation using PT-specific *Twist1* knockout (PT-*Twist1*^–/–^) and littermate control (PT-*Twist1*^+/+^) murine models subjected to UUO and unilateral ischemia–reperfusion injury (UIRI). Histopathological evaluation demonstrated that *Twist1* ablation attenuated indices of tubular injury, including interstitial collagen deposition, inflammatory infiltration, and architectural distortion, in both disease models (Fig. 1a and Fig. S1b), consistent with established fibrotic mechanisms. Mechanistic interrogation integrating transcriptomic profiles from the PT-specific *Twist1* knockout UUO murine dataset (GSE60685) [15] and knockout models revealed the predominance of glycolytic pathways, as evidenced by coordinated dysregulation of metabolic genes and enrichment of FAO/glycolysis signatures (Fig. 1b-e). Metabolomic profiling further confirmed reduced lactate accumulation in the kidneys of PT-*Twist1*^–/–^ mice after UIRI (Fig. 1f and Fig. S2), implicating lactate-mediated signaling in tubular reprogramming. Complementary in vitro studies demonstrated the direct regulation of glycolytic enzymes and dedifferentiation markers by *TWIST1* in TECs (Fig. 1g). These findings support a paradigm in which tubular Twist1 orchestrates metabolic switching to link acute injury with chronic fibrotic progression.

### Glycolytic persistent activation and lactate accumulation within tubular maladaptation and fibrotic progression in kidney injury

To investigate lactate metabolism in kidney Injury pathogenesis, glycolytic reprogramming was systematically analyzed across clinical and experimental models. Baseline demographic and clinical characteristics of patients in the Nephroseq database and those from Xijing Hospital are provided in Table S1 and Table S2, respectively. Initial interrogation of Nephroseq datasets comparing CKD tubulointerstitial profiles with BioChain-derived normal kidney RNA revealed marked upregulation of the rate-limiting glycolytic enzyme *LDHA* in kidney RNA from 48 patients with CKD compared with that from 5 healthy individuals (Fig. S3a). This finding was corroborated in renal tissues from 23 patients with IgA nephropathy (IgAN) and paracancerous renal tissues from 13 patients with renal cell carcinoma who underwent nephrectomy at Xijing Hospital between 2020 and 2023. IgAN was the predominant CKD etiology in both the present cohort and China. Both *LDHA* and *HK2* expression levels were significantly elevated in IgAN-affected kidneys relative to paracancerous controls (Fig. S3b-c), further supporting dysregulated glycolysis during CKD progression. Subsequent cross-species RNA-sequencing analyses of biopsies from patients with IgAN and kidneys from murine UIRI and UUO models were conducted to delineate conserved metabolic pathways.

**Fig. 2:**
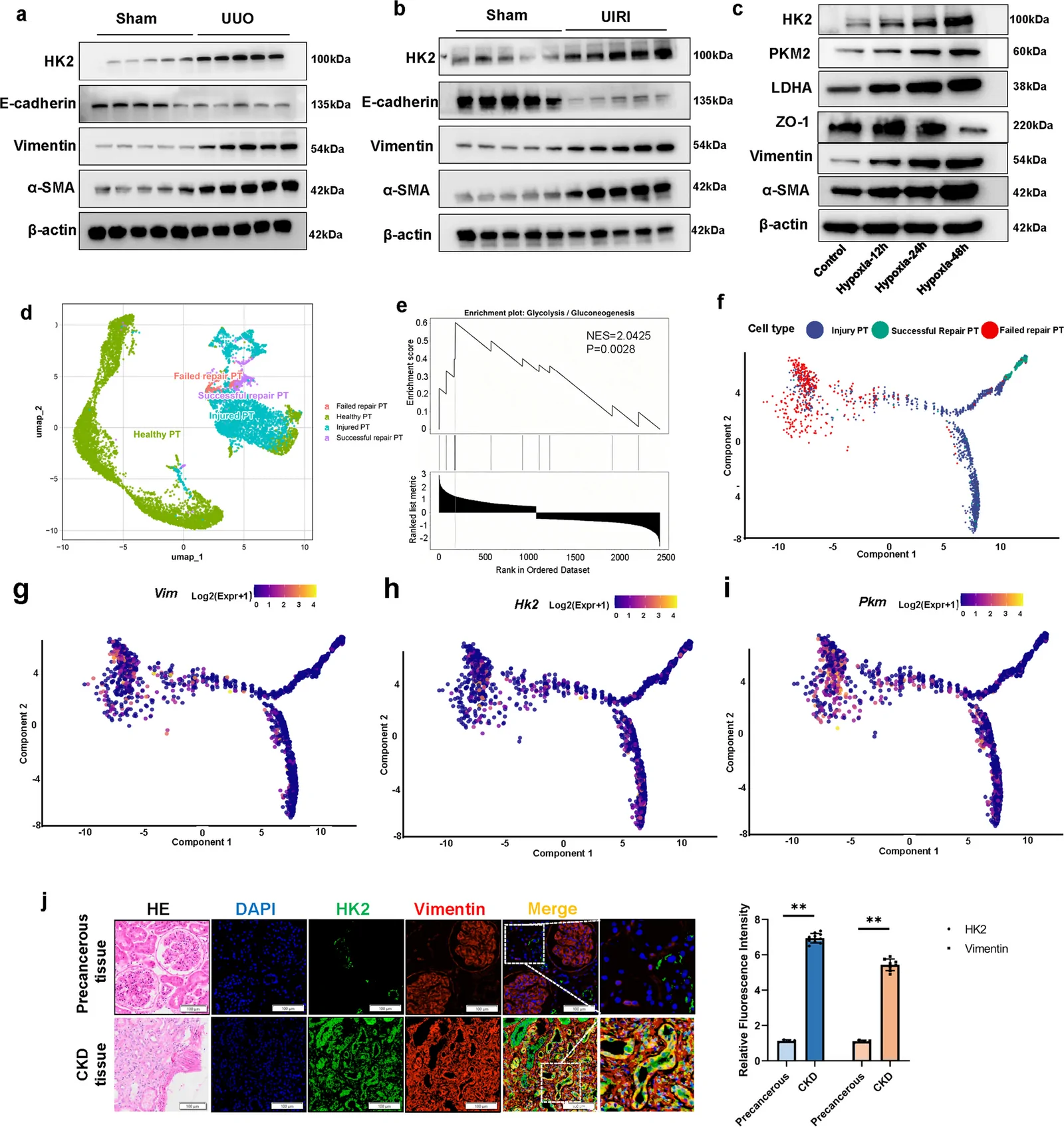
Glycolytic metabolic reprogramming drives tubular dedifferentiation and maladaptive repair in kidney injury across murine models and human CKD. **a** Western blotting analysis of vimentin, HK2, α-SMA, and E-cadherin expression in tubular epithelial cells (TECs) from UUO kidneys (n = 5 per group). **b** Western blotting analysis of vimentin, HK2, α-SMA, and E-cadherin expression in TECs from UIRI kidneys (n = 5 per group). **c** Western blotting analysis of key glycolytic metabolic proteins and dedifferentiation markers in hypoxia-treated HK-2 cells (n = 3). **d** UMAP visualization of proximal tubule cell subpopulations. **e** Gene set enrichment analysis showing enrichment of differentially expressed genes in the glycolysis/gluconeogenesis pathway in failed-repair PT cells (NES = 2.0425, *P* = 0.0027701). **f** Trajectory analysis of PT cell subpopulations. Green indicates failed-repair PT cells, blue indicates successful-repair PT cells, and red indicates injured PT cells. Expression and distribution of *Vim* (**g**), *Hk2* (**h**), and *Pkm* (**i**) in PT cell subpopulations. Color intensity from blue to red indicates increasing expression levels. **j** H&E and immunofluorescence staining of kidney tissues from patients with CKD caused by IgA nephropathy (IgAN) and controls (adjacent non-tumor kidney tissues). Magnification, × 20. Red boxes indicate enlarged regions. Quantification of relative fluorescence intensity is shown. **P < 0.01, CKD group, n = 10; control group, n = 4

The metabolic basis of renal fibrogenesis was systematically delineated through cross-species transcriptomic interrogation. Comparative analysis of CKD and control renal tissues revealed preferential enrichment of genes associated with glycolysis/gluconeogenesis (Fig. S3d). This pattern was conserved in murine UUO and UIRI models, in which coordinated upregulation of glycolytic regulators was observed at 14 days after injury (Fig. S4a,c), with pathway-level confirmation by KEGG enrichment analysis (Fig. S4b,d). Protein-level validation demonstrated concomitant elevation of the glycolytic gatekeeper HK2 and tubular dedifferentiation markers in whole fibrotic kidney tissues (Fig. S4e-f) and TECs isolated from fibrotic kidneys (Fig. 2a-b). Notably, in vitro hypoxia models recapitulated this metabolic–epithelial plasticity axis through the synchronized modulation of glycolytic and dedifferentiation markers (Fig. 2c), establishing progressive glycolytic activation as a hallmark of fibrotic progression.

This study further elucidated the metabolic determinants underlying TEC fate specification during fibrotic repair by employing single-cell (Sc) transcriptomic analysis. ScRNA-seq profiling of murine kidneys (Sham, UIRI-Day3, UIRI-Day14) identified 21 distinct cellular clusters (Fig. S5a), with PT subpopulations classified into Healthy, Injured, and Failed Repair phenotypes based on established biomarkers (Fig. 2d). Compared to both the healthy group and the successfully repaired group, Failed Repair PT cells and Injured PT cells exhibited markedly elevated expression of key glycolytic enzyme genes, including *Hk2*, *Pfkp*, *Slc2a1*, and *Pkm* (Fig. S5b). Additionally, Failed Repair PT cells showed significant dysregulation of FAO and glycolytic pathway components relative to Successful Repair PT cells (Fig. S5c-d and Fig. 2e). Pseudotemporal trajectory analysis positioned Injured PT cells at the root of a bifurcating differentiation path leading to Successful (right branch) or Failed Repair (left branch) terminal states (Fig. S5e-f and Fig. 2f), with the Failed Repair population exhibiting co-enrichment of glycolytic effectors such as *Hk2* and *Pkm* alongside mesenchymal markers such as *Vim* at the branch termini (Fig. 2g-i). Complementary immunofluorescence analysis of CKD patient biopsies further revealed spatial co-localization of the glycolytic enzyme HK2 with the maladaptive repair marker vimentin in tubular cytoplasm (Fig. 2j). Collectively, these multi-dimensional findings-spanning human CKD biopsies, in vivo murine fibrosis models, the sc transcriptomic dissection of PT cell fate, and in vitro mechanistic validation-establish glycolytic reprogramming as a central regulatory axis driving TEC maladaptive repair and the progression of renal fibrosis.

**Fig. 3:**
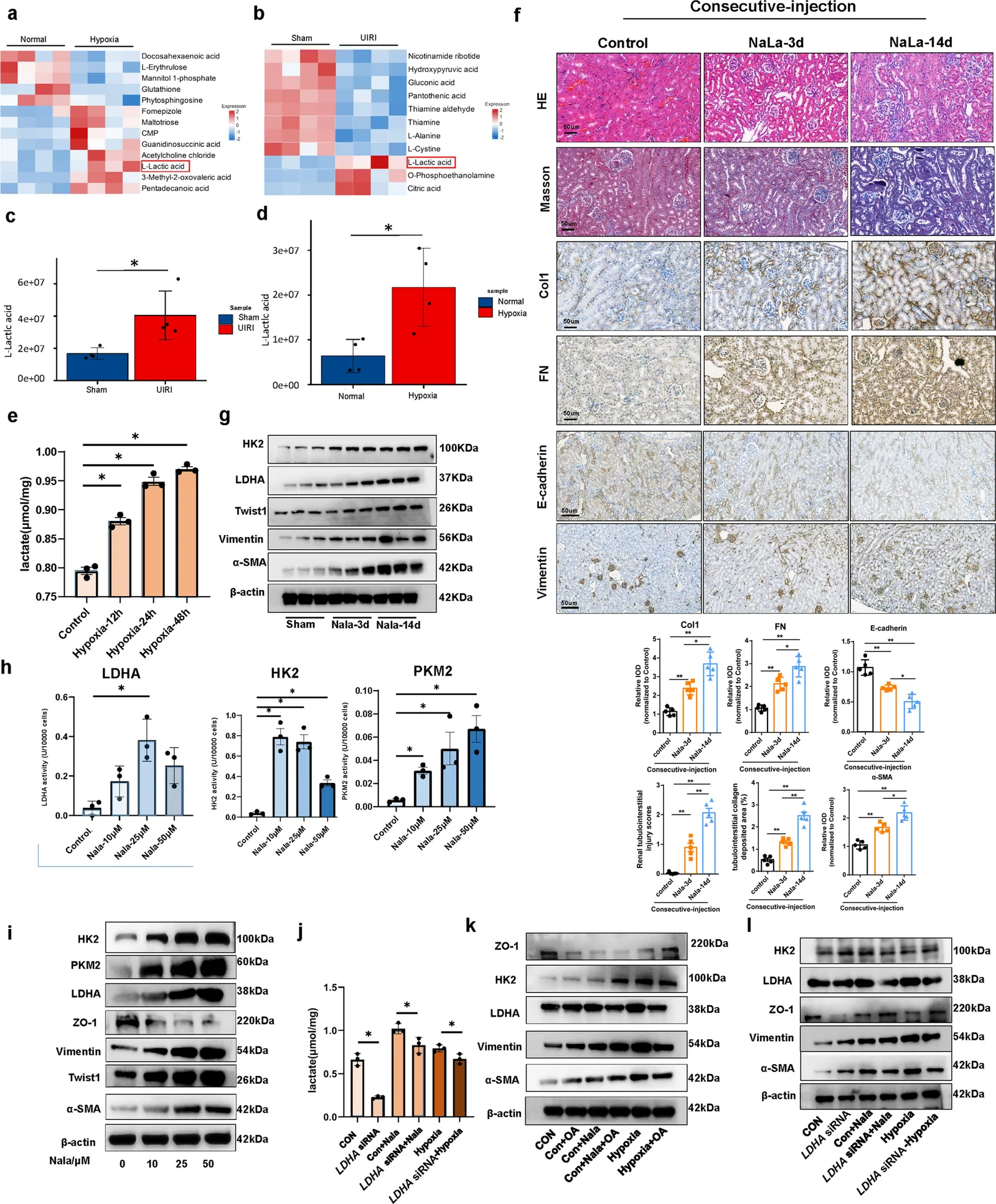
Lactate-driven glycolytic reprogramming exacerbates renal fibrosis and tubular injury in murine and cellular kidney injury models. **a** Heatmap of selected differentially abundant metabolites identified by metabolomic analysis in HK-2 cells cultured under hypoxic and normoxic conditions. **b** Heatmap of selected differentially abundant metabolic intermediates in kidney tissues from sham and UIRI mice. **c** Lactate concentration in kidney tissues from sham and UIRI mice. *P < 0.05, n = 4. **d** Relative lactate levels in hypoxic and control HK-2 cells. *P < 0.05, n = 4. **e** Lactate concentration in the supernatants of hypoxic HK-2 cells. *P < 0.05, n = 3. **f** H&E, Masson, and immunohistochemical staining of fibronectin (FN), type I collagen (Col1), vimentin, and E-cadherin in kidney tissues from sham, sodium lactate (NaLa)-treated for 3 days, and NaLa-treated for 14 days mice. Magnification, × 20. *P < 0.05, **P < 0.01, n = 5. **g** Western blotting analysis of HK2, LDHA, vimentin, E-cadherin, Twist1, and α-SMA in UIRI mice treated with intraperitoneal NaLa for 3 or 14 days (n = 3). **h** Activities of LDHA, HK2, and PKM2 in HK-2 cells treated with different concentrations of NaLa. *P < 0.05, n = 3. **i** Western blotting analysis of ZO-1, HK2, LDHA, PKM2, vimentin, α-SMA, and Twist1 in HK-2 cells treated with increasing concentrations of exogenous NaLa (n = 3). **j** Lactate concentration in the supernatants of HK-2 cells treated with hypoxia and exogenous NaLa following LDHA siRNA treatment. *P < 0.05, n = 3. **k** Western blotting analysis of ZO-1, LDHA, HK2, α-SMA, and vimentin in HK-2 cells treated with oxamate (OA), hypoxia, and exogenous NaLa (n = 3). **l** Western blotting analysis of ZO-1, LDHA, HK2, α-SMA, and vimentin in HK-2 cells treated with hypoxia and exogenous NaLa following LDHA siRNA treatment (n = 3). Con, control group

The pathological consequences of glycolytic flux amplification in injured TECs were investigated through integrated metabolomic profiling. Non-targeted metabolomics of UIRI murine kidneys and hypoxia-exposed HK-2 cells demonstrated broad metabolic perturbations, with substantial accumulation of lactate, the terminal metabolite of glycolysis, observed in both in vitro and in vivo systems (Fig. 3a-b and Fig. S6a-b). Intracellular lactate levels were quantified in UIRI kidney tissues and hypoxia-treated HK2 cells using a lactate assay kit, revealing a significant increase in lactate levels following UIRI surgery and hypoxia exposure, consistent with the metabolomic findings (Fig. 3c-d). Furthermore, extracellular lactate measurement demonstrated a hypoxia duration-dependent escalation (Fig. 3e), suggesting a spatiotemporal correlation between glycolytic activation and lactate dynamics.

### Lactate serves as a metabolic checkpoint driving glycolysis-fibrosis axis in renal tubules

Whether a causal relationship exists between glycolytic hyperactivity and fibrotic progression was investigated through pharmacological perturbation of tubular metabolism. Administration of the glycolytic inhibitor 2-DG significantly attenuated UUO/UIRI-induced renal fibrosis, as evidenced by histopathological reductions in collagen deposition, inflammatory infiltration, and tubular injury (Fig. S7a-b). Immunohistochemical analysis revealed 2-DG-mediated molecular reprogramming in tubular epithelia, characterized by restoration of E-cadherin and suppression of α-SMA (Fig. S8a-b). Complementary molecular profiling confirmed coordinated downregulation of fibrotic mediators at both the transcriptional (Fig. S8c) and translational levels (Fig. S8d), mechanistically linking glycolytic inhibition to antifibrotic outcomes. These findings identify lactate-driven glycolytic flux as an actionable metabolic checkpoint governing the fibrogenic transition of TECs.

Lactate’s causal involvement in renal fibrotic progression was directly interrogated through pharmacological induction of systemic lactate elevation. Intraperitoneal administration of sodium lactate (NaLa) elicited time-dependent modulation of TEC plasticity: transient exposure (3 days) initiated dedifferentiation programs and interstitial injury, whereas chronic treatment (14 days) drove progressive fibrotic transformation, as evidenced by histopathological changes in collagen deposition (type I collagen), matrix remodeling (fibronectin), and epithelial–mesenchymal transition, along with downregulated E-cadherin and upregulated vimentin (Fig. 3f). Mechanistically, NaLa challenge enhanced glycolytic output in TECs, upregulating some key metabolic mediators and some maladaptive repair effectors (Fig. 3g-h), thereby positioning lactate as both a metabolic byproduct and a pathogenic driver in fibrogenic circuitry.

Lactate’s regulatory hierarchy in TEC metabolic remodeling was mechanistically dissected through pharmacological and genetic modulation. Gradient exposure of HK-2 cells to NaLa induced a concentration-dependent increase in glycolytic effectors (HK2, PKM2, and LDHA), accompanied by mesenchymal transition, upregulated vimentin and α-SMA, and suppression of the epithelial marker zonula occludens-1 (ZO-1) (Fig. 3i), demonstrating the dual role of lactate in fueling glycolysis and driving dedifferentiation. Pharmacological inhibition with oxamate and genetic inhibition using *LDHA* siRNA (Fig. 3j) abolished hypoxia/NaLa-induced glycolytic activation (HK2 and LDHA) and mesenchymal reprogramming, accompanied by downregulated vimentin and α-SMA and upregulated ZO-1 (Fig. 3k-l), establishing lactate as both a mediator and an amplifier of hypoxia-triggered metabolic–structural coupling in TECs.

### Twist1 serves as a transcriptional master switch for glycolytic activation in fibrotic tubules

**Fig. 4:**
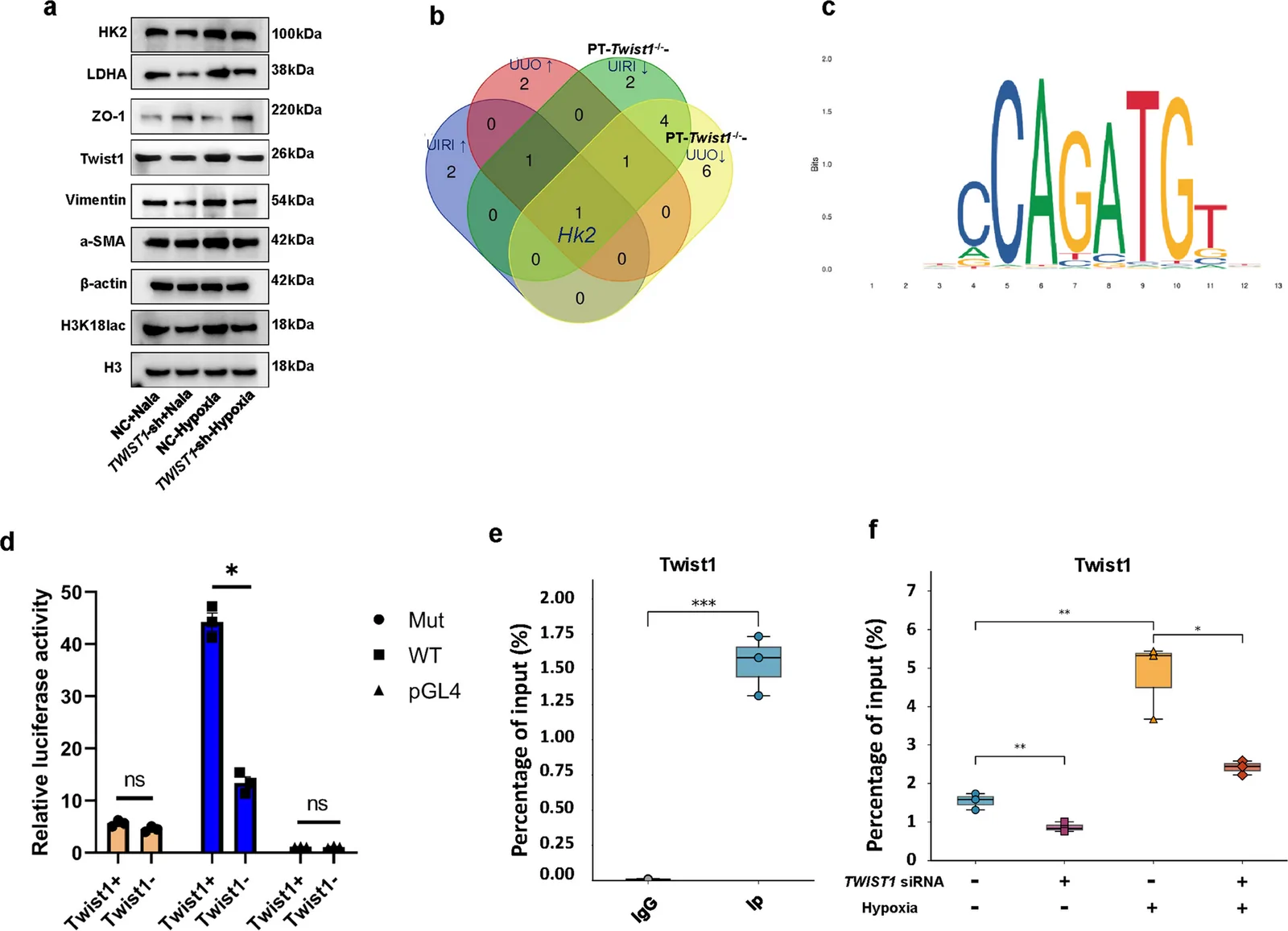
Twist1 transcriptionally activates HK2 to promote glycolytic reprogramming and tubular dedifferentiation under hypoxia. **a** Western blotting analysis of dedifferentiation markers and key glycolytic enzymes in hypoxia-treated HK-2 cells following Twist1 inhibition (n = 3). **b** Transcriptomic comparison across multiple mouse models identified HK2 as the only commonly upregulated differentially expressed gene. **c** Predicted Twist1-binding sites within the *HK2* promoter identified using the JASPAR transcription factor database. **d** Dual-luciferase reporter assay validating the transcriptional regulation of the *Hk2* promoter by Twist1. pGL4 represents the Promega E8421 vector. *P < 0.05, n = 3. **e** ChIP-PCR analysis of the indicated *HK2* promoter regions using anti-Twist1 antibodies in HK-2 cells, comparing immunoprecipitated (IP) samples with negative controls. ***P < 0.001, n = 3. **f** ChIP-PCR analysis of the indicated *HK2* promoter regions using anti-Twist1 antibodies in HK-2 cells from the control, *TWIST1* siRNA, hypoxia, and hypoxia & *TWIST1* siRNA groups. *P < 0.05, **P < 0.01, n = 3

Twist1’s hierarchical regulation of glycolytic reprogramming in renal fibrosis was delineated through molecular perturbation and transcriptional validation. Genetic silencing of *TWIST1* via shRNA attenuated hypoxia/NaLa-induced glycolytic activation, along with downregulated HK2 and LDHA and mesenchymal transition in TECs (Fig. 4a), positioning Twist1 as a nodal regulator of metabolic–structural coupling. Cross-model transcriptomic analysis of *Twist1* knockout mice subjected to UUO/UIRI revealed conserved dysregulation of glycolytic hubs, including *Hk2* (Fig. 4b), prompting mechanistic investigation of Twist1’s transcriptional capacity. Computational prediction using the JASPAR database identified putative Twist1-binding motifs within the *HK2* promoter region (Fig. 4c), which were functionally validated by dual-luciferase assays demonstrating Twist1-dependent transcriptional activation of HK2 (Fig. 4d). ChIP-qPCR confirmed the presence of a Twist1-binding motif in the *HK2* promoter and demonstrated that hypoxia substantially facilitated Twist1 binding to the *HK2* promoter region (Fig. 4e-f and Fig. S9; site 177–186 +). These findings establish Twist1 as a master transcriptional regulator of tubular glycolysis through direct engagement of the *Hk2* promoter, mechanistically linking epithelial plasticity to metabolic rewiring during fibrotic progression.

### Histone lactylation at H3K18 licenses a lactate-twist1 feedback loop to drive fibrotic metabolic reprogramming

**Fig. 5:**
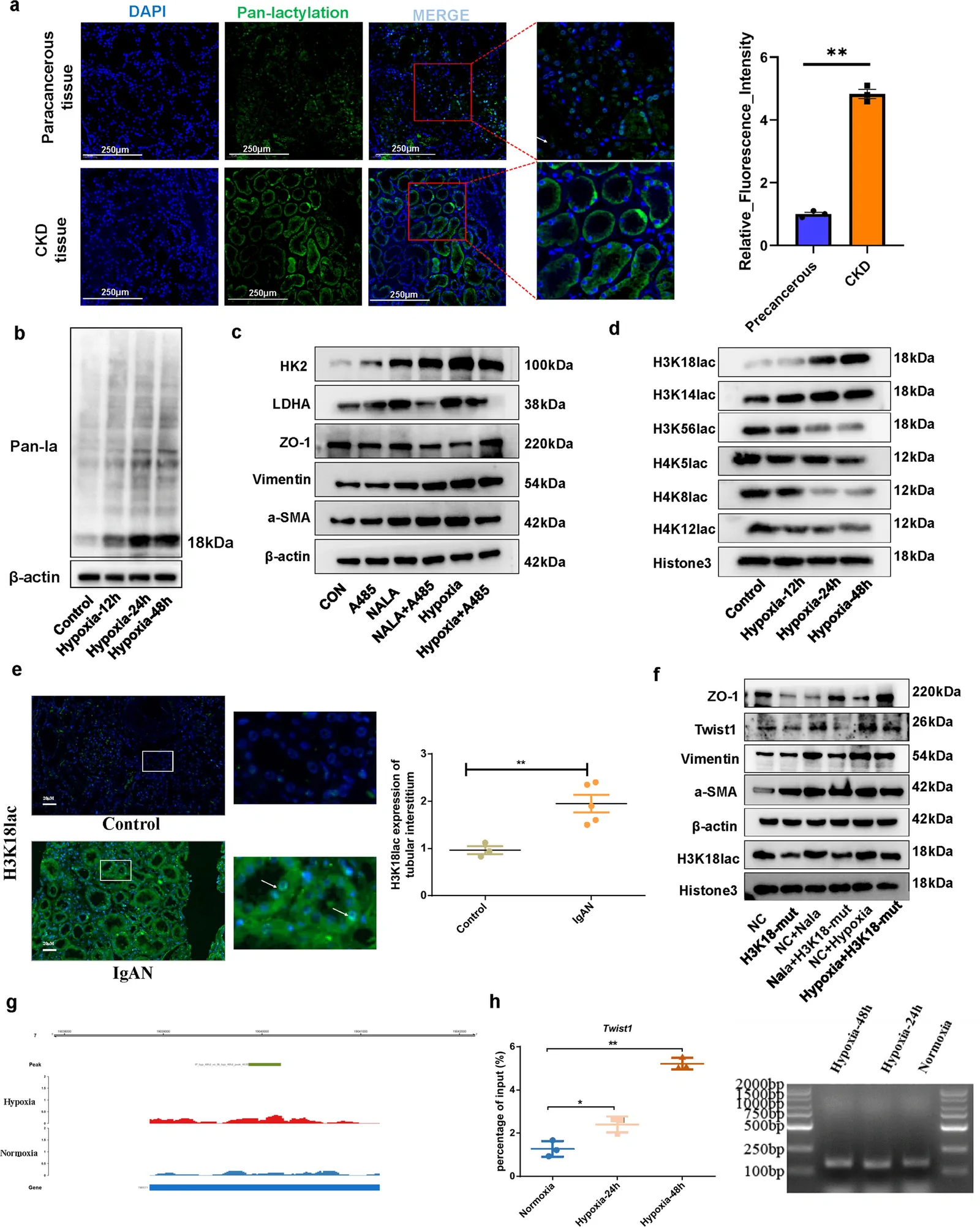
H3K18 is a key lactylation site that enhances glycolysis and dedifferentiation of TECs. **a** Immunofluorescence staining of pan-lactylation in CKD and adjacent non-tumor kidney tissues. Magnification, × 20. The region outlined by the red box is shown at higher magnification. Quantification of fluorescence intensity is shown. **P < 0.01, n = 3. **b** Western blotting analysis showing pan-lactylation levels in renal TECs following different durations of hypoxia (n = 3). **c** Western blotting analysis of ZO-1, LDHA, HK2, α-SMA, and vimentin in HK-2 cells treated with A485, hypoxia, and exogenous NaLa in vitro (n = 3). **d** Western blotting analysis of histone H3 and H4 lactylation at different lysine residues in HK-2 cells following different durations of hypoxia (n = 3). **e** Immunofluorescence staining of H3K18lac in CKD (n = 5) and adjacent non-tumor kidney tissues (n = 3). Magnification, × 20. The boxed region is shown at higher magnification. Quantification of fluorescence intensity is shown. **P < 0.01. **f** Following H3K18 mutation in HK-2 cells, Western blotting analysis was performed to evaluate the effects of hypoxia and exogenous NaLa on the expression of ZO-1, H3K18lac, Twist1, vimentin, and α-SMA (n = 3). **g** Genome browser tracks of ChIP-seq signals at representative target genes. Red and blue rectangles indicate H3K18lac peak regions on target gene promoters. **h** ChIP-PCR analysis of the indicated promoters using anti-H3K18lac antibodies in HK-2 cells. *P < 0.05, **P < 0.01, n = 3

The epigenetic dimension of lactate signaling in CKD pathogenesis was delineated through global lactylation profiling. Pan-protein lactylation immunofluorescence revealed pronounced lactylation accumulation in TECs from patients with IgAN, particularly within nuclear compartments, compared with paracancerous controls (Fig. 5a), indicating the spatial reorganization of lactate-mediated epigenetic regulation during CKD progression.

The functional hierarchy of histone lactylation (Kla) in coordinating tubular metabolic–phenotypic coupling was investigated through epigenetic perturbation. Hypoxic exposure induced a time-dependent increase in global lactylation in HK-2 cells, with marked enrichment of lactylated nuclear proteins at 15–20 kDa, corresponding to histone isoforms (Fig. 5b). Pharmacological inhibition of the epigenetic writer enzyme p300/CBP using A485 attenuated hypoxia/NaLa-induced glycolytic activation and mesenchymal transition (Fig. 5c), while paradoxically enhancing these pathways under basal conditions, indicating the bidirectional role of Kla in regulating tubular homeostasis.

Hypoxia-sensitive Kla signatures governing tubular epithelial reprogramming were identified through lysine-residue mapping. Histone fractionation revealed time-dependent enrichment of lactylation at histone H3 lysine 18 (H3K18lac) during hypoxic progression (Fig. 5d). Spatial validation further demonstrated pronounced H3K18lac accumulation in TECs from patients with CKD compared with controls (Fig. 5e), establishing this epigenetic mark as a nodal modification during hypoxic injury.

The epigenetic regulation of metabolic-phenotypic coupling by H3K18lac was mechanistically defined through site-directed mutagenesis and chromatin interactome mapping. Lysine-to-arginine substitution at H3K18 attenuated hypoxia/NaLa-induced expression of mesenchymal effectors in TECs (Fig. 5f), establishing this lactylation site as a nodal epigenetic switch. Subsequent ChIP-seq analysis revealed hypoxia-induced H3K18lac enrichment at the promoters of fibro-metabolic regulators, particularly those involved in transforming growth factor-β signaling and glycolysis/gluconeogenesis pathways (Fig. S10a). Twist1 emerged as the most prominently upregulated transcriptional hub (Fig. S10b), and direct H3K18lac occupancy at its promoter region was validated by ChIP-seq (Fig. 5g) and ChIP-qPCR (Fig. 5h). Mechanistically, the H3K18R mutation abolished hypoxia/NaLa-induced Twist1 upregulation (Fig. 5f), completing an epigenetic-metabolic feedback loop in which lactate-derived histone lactylation licenses Twist1 transcription to perpetuate glycolytic reprogramming. To explore the temporal window during which the lactate-Twist1 regulatory loop becomes irreversible, animal experiments involving 2-DG administration at different stages were conducted. One group received 2-DG from days 1 to 7 after surgery, whereas the other received 2-DG from days 8 to 14 postoperatively. Notably, the group treated at the later stage exhibited more pronounced improvements in glycolytic and fibrotic markers (Fig. S11).

### Serum lactate as a bidirectional sentinel: prognostic stratifier in AKI and causal driver of CKD trajectory

**Fig. 6:**
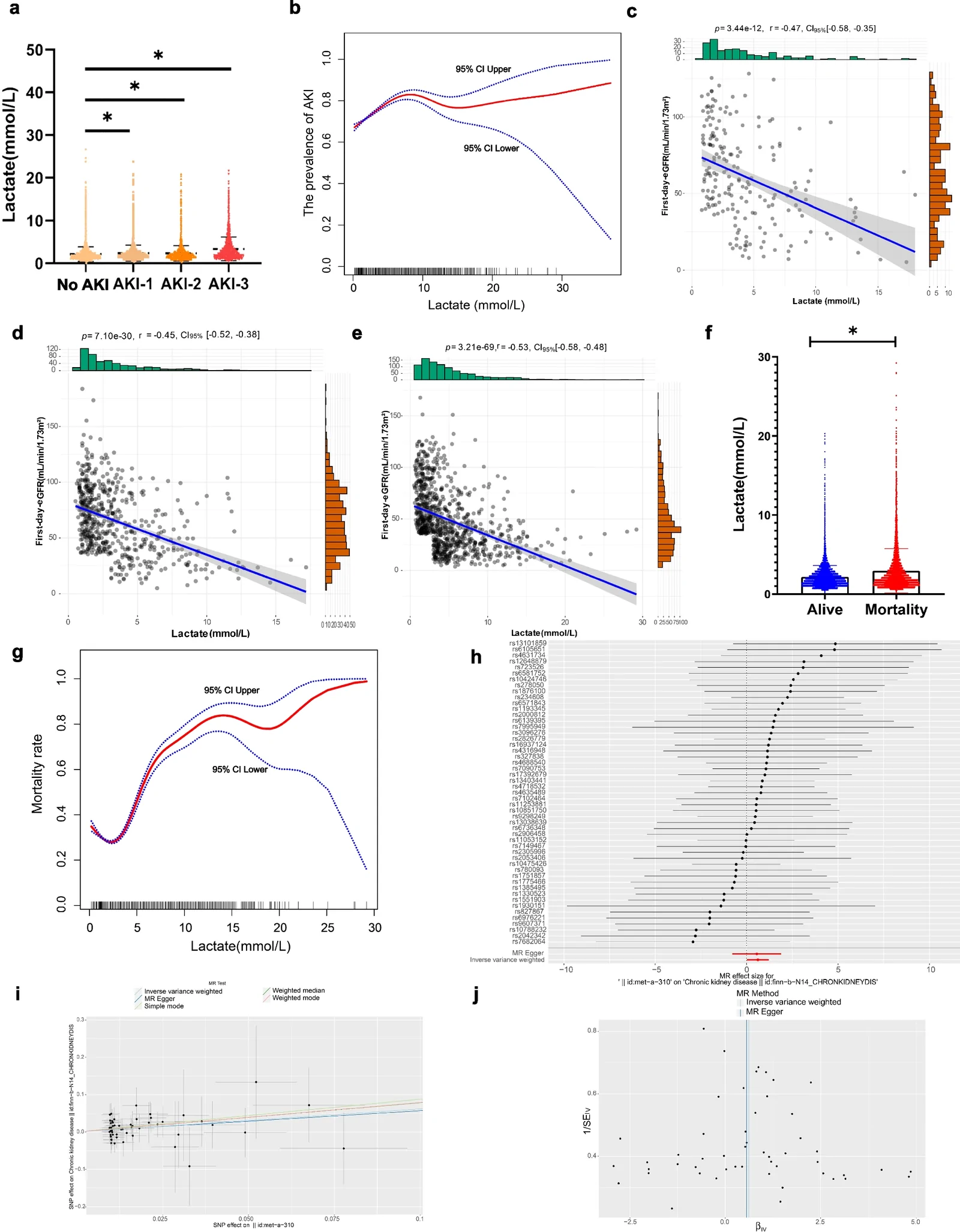
Serum lactate level is a potential predictor of AKI prognosis and CKD. **a** Mean serum lactate concentration on ICU admission was compared between patients who developed AKI (stages 1, 2, or 3) and those who did not. Statistical significance is indicated by * (all *P* < 0.05). Total sample size, n = 20,936. **b** Smooth-curve fitting using a generalized additive model revealed a nonlinear relationship between serum lactate level and AKI prevalence in the MIMIC database. The red solid line represents the fitted curve, and the blue lines indicate the 95% confidence interval (CI). **c** Scatter plot showing the correlation between mean serum lactate concentration and eGFR in patients with stage 1 AKI who died during their ICU stay. Pearson’s r = − 0.47, *P* < 0.05. **d** Scatter plot showing the correlation between mean serum lactate concentration and eGFR in patients with stage 2 AKI who died during their ICU stay. Pearson’s r = − 0.45, *P* < 0.05. **e** Scatter plot showing the correlation between mean serum lactate concentration and eGFR in patients with stage 3 AKI who died during their ICU stay. Pearson’s r = − 0.53, *P* < 0.05. **f** Mean serum lactate concentration on the first day of ICU admission was significantly higher in patients who died than in those who survived. *P* < 0.05. **g** Smooth-curve fitting using a generalized additive model revealed a nonlinear relationship between serum lactate level and ICU mortality. **h** Mendelian randomization analysis based on genome-wide association studies supported a causal role of lactate in CKD development (odds ratio = 1.854, *P* < 0.05). **i** Scatter plot assessing the causal association between lactate and CKD. **j** Funnel plot evaluating horizontal pleiotropy in the causal association between lactate and CKD

The prognostic relevance of serum lactate levels in AKI was assessed through multidimensional analyses of critical care cohorts using the MIMIC-IV v1.0 database. Critically ill patients were stratified into AKI severity cohorts according to KDIGO criteria based on dynamic monitoring of creatinine levels and urine output (Fig. S12). Elevated systemic lactate levels at ICU admission were progressively associated with AKI stage, with initial lactate concentrations increasing stepwise across advancing AKI severity categories (Fig. 6a). Nonparametric regression analysis revealed a positive monotonic relationship between lactate levels at ICU admission and AKI incidence (Fig. 6b), whereas inverse correlations were observed between eGFR and lactate concentrations within each AKI stratum (Fig. 6c-e). Mortality risk exhibited a nonlinear threshold response to elevated lactate levels, with critical inflection points identified below conventional diagnostic thresholds for hyperlactatemia (Fig. 6f-g), establishing serum lactate as a sensitive prognostic biomarker for renal outcomes in critically ill patients.

A causal relationship between systemic lactate homeostasis and CKD progression was explored using bidirectional Mendelian randomization (MR) analysis. Building on the acute-phase findings, two-sample MR analysis employing genome-wide significant lactate-associated single-nucleotide polymorphisms demonstrated a statistically significant positive association between genetically predicted serum lactate levels and CKD risk (Fig. S13 and Fig. 6h). MR-Egger regression intercept analysis confirmed minimal horizontal pleiotropy, whereas funnel plot symmetry supported the robustness of the instrumental-variable assumptions (Fig. 6i-j). Complementary MR-PRESSO outlier correction preserved the direction of the causal association. In reverse MR analysis, genetically predicted CKD risk showed no significant positive causal effect on circulating lactate levels using the primary inverse-variance weighted method (β = 0.0428, SE = 0.0337, *P* = 0.204), ruling out substantial reverse causation between CKD progression and lactate dysregulation. These findings establish serum lactate as a modifiable risk factor that orchestrates the transition from acute to chronic renal injury.

## Discussion

Renal fibrosis, characterized by tubular atrophy and excessive extracellular matrix deposition, is a hallmark of the transition from AKI to CKD [16]. It represents a common final pathological pathway for virtually all forms of progressive CKD, irrespective of the initial inciting insults, including immune-mediated glomerulonephropathies (such as IgAN, the primary clinical cohort in this study), ischemic kidney injury, obstructive nephropathy, diabetic kidney disease, and hypertensive nephrosclerosis. Despite diverse pathogenic triggers, maladaptive repair of TECs constitutes the central convergent cellular event driving the development and progression of renal tubulointerstitial fibrosis. Notably, metabolic reprogramming, particularly dysregulation of glycolytic and FAO programs in TECs, has increasingly been recognized as a pivotal molecular mechanism underlying impaired TEC repair homeostasis across different CKD etiologies. Previous work revealed the role of Twist1 in lipid accumulation through FAO suppression [6]. The present study extends this paradigm by showing that Twist1 orchestrates a dual metabolic insult involving FAO suppression and glycolytic hyperactivation. This dual role highlights the versatility of Twist1 as a master metabolic regulator capable of rewiring TEC processes to favor a profibrotic phenotype. Notably, in cardiac fibrosis, Twist1 promotes fibroblast activation through transforming growth factor-β signaling, suggesting the existence of tissue-specific regulatory networks [17]. Previous work from our group demonstrated that harmine, a specific Twist1 inhibitor, attenuated renal fibrosis in UIRI and UUO models [18]. Central to this process is the maladaptive repair of TECs, which undergo metabolic reprogramming from FAO to glycolysis-a shift that perpetuates dedifferentiation and fibrogenesis [19, 20].

Hypoxia is a hallmark of AKI and CKD and drives TECs toward glycolytic dependence [21]. Hypoxia was shown to directly upregulate Twist1 in TECs, both in vivo (UIRI/UUO models) and in vitro (hypoxic HK-2 cells). This upregulation was associated with increased expression of glycolytic rate-limiting enzymes, including HK2 and LDHA, as well as mesenchymal markers, including α-SMA and vimentin [6]. These findings are consistent with previous observations in cancer biology, in which Twist1 enhances glycolysis by upregulating HK2 and glucose transporters [22]. However, the present study uniquely identifies Twist1 as a hypoxia-responsive transcription factor in renal fibrosis, bridging metabolic dysregulation and structural injury.

RNA-seq and ChIP-seq analyses identified HK2 as a direct transcriptional target of Twist1. Dual-luciferase assays confirmed Twist1 binding to conserved motifs within the *HK2*promoter, whereas genetic ablation of Twist1 in TECs suppressed HK2 expression and lactate production. These findings establish a positive feedback loop in which hypoxia-induced Twist1 activates HK2-driven glycolysis, thereby amplifying lactate accumulation, which in turn stabilizes Twist1 expression through epigenetic modifications [23, 24]. Notably, this loop resembles mechanisms reported in cancer metastasis but differs in its tissue-specific outcome [25], resulting in fibrosis rather than tumor progression. Beyond this tubular cell-autonomous regulatory axis, TEC-derived lactate may also function as a key paracrine signaling molecule that modulates the profibrotic phenotypes of neighboring pericytes, fibroblasts, and infiltrating immune cells within the renal microenvironment. This paracrine regulatory effect may represent an important synergistic mechanism underlying the robust antifibrotic phenotype observed in PT-specific *Twist1* knockout mice.

Lactate, once considered a waste product of glycolysis, is now recognized as a key signaling molecule [26]. In CKD, elevated lactate levels were observed in TECs and serum and correlated with fibrosis severity [27]. These findings are consistent with studies linking lactate to fibroblast activation and cardiac surgery-associated AKI [28, 29], while uniquely implicating lactate in autocrine TEC reprogramming.

Lactate serves as a substrate for histone lactylation, a recently identified post-translational modification [30, 31]. In hypoxic TECs, H3K18 lactylation (H3K18lac) was identified as the predominant histone lactylation mark and was enriched at the Twist1 promoter [23, 24]. ChIP-seq and ChIP-qPCR analyses further confirmed that H3K18lac directly promotes Twist1 transcription, thereby closing a metabolic-epigenetic positive-feedback loop [13, 32]. This mechanism is consistent with observations in hepatic stellate cells, in which lactate-induced histone lactylation promotes collagen synthesis [23], but the present findings extend this concept to TEC-autonomous fibrotic reprogramming. Our study systematically delineates the regulatory contribution of metabolic reprogramming to the AKI-to-CKD transition through H3K18 lactylation, consistent with emerging evidence that lactylation is a key molecular mechanism in this transition. Beyond the H3K18lac-centered mechanism examined here, accumulating evidence indicates that both histone and non-histone lactylation exert important regulatory effects in renal fibrosis. For example, lactate accumulation driven by the glycolytic enzyme PFKFB3 markedly increases histone H4K12 lactylation (H4K12la). H4K12la is enriched at the promoter regions of core NF-κB pathway genes, thereby transcriptionally activating inflammatory cascades and amplifying tubular inflammation and fibrotic progression [32]. Non-histone lactylation also acts as a critical mediator of renal injury. Lactylation of aldehyde dehydrogenase 2 (ALDH2) at K52 promotes ubiquitin–proteasome-mediated degradation of the mitophagy receptor PHB2, inhibiting mitophagy and exacerbating mitochondrial dysfunction in renal tubular cells. This pathogenic effect can be reversed by SIRT3, which functions as an ALDH2 delactylase [8]. Furthermore, lactylation of citrate synthase (CS) at K370 suppresses its enzymatic activity, disrupts mitochondrial homeostasis, and activates the NLRP3 inflammasome, ultimately promoting the AKI-to-CKD transition [33].

Although lactate can also contribute to protein acetylation through acetyl-CoA synthesis, previous experiments excluded acetylation as the principal mechanism underlying Twist1 activation [34, 35]. Hypoxia increased global protein acetylation; however, exogenous lactate did not further augment acetylation, supporting the specificity of lactylation in this context. This finding contrasts with sepsis-associated AKI, in which hyperacetylation of pyruvate dehydrogenase E1 subunit alpha 1 exacerbates lactate production [13], highlighting the disease and context-dependent roles of lactate-associated post-translational modifications.

Contrary to the expectation that early glycolytic blockade would provide greater antifibrotic protection, delayed 2-DG intervention produced more pronounced reductions in glycolytic and fibrotic markers. This seemingly paradoxical result suggests that glycolytic activity has distinct functions at different stages following AKI. During the early phase (days 1–7), glycolysis in TECs may represent an adaptive and largely reversible metabolic response that supports cell survival and reparative proliferation. Premature suppression of this adaptive glycolysis by 2-DG may compromise endogenous repair and consequently limit its net antifibrotic benefit. In contrast, during the later phase (days 8–14), the lactate/H3K18lac/Twist1/HK2 positive-feedback loop may become increasingly established and self-sustaining, transforming glycolysis into a maladaptive driver of fibrotic reprogramming. At this stage, continued glycolytic flux is likely required to maintain the fibrogenic circuit; thus, its interruption by 2-DG produces a more substantial therapeutic effect. These findings suggest that the shift from adaptive to pathogenic glycolysis occurs within a progressive commitment window. Once the lactate-Twist1 circuit becomes the dominant driver of fibrotic remodeling, it may represent both a critical transition point in disease progression and a window of heightened therapeutic vulnerability. Therefore, the optimal timing of glycolytic inhibition during AKI-to-CKD progression may be after the early reparative phase, when the lactate-Twist1 axis has matured into a major driver of fibrotic reprogramming.

In the MIMIC-IV cohort (n = 20,936), serum lactate levels were inversely correlated with eGFR and predicted AKI progression and mortality [36–38]. Patients with lactate levels > 7 mM at ICU admission exhibited a 2.1-fold higher risk of CKD, consistent with previous critical care studies [38]. Importantly, our MR analysis extends these observational findings by providing genetic evidence supporting a causal role of elevated lactate in CKD risk, representing a novel contribution to the field [39].

Although our study provides valuable insights, several limitations should be acknowledged. First, the absence of serum lactate measurements in a dedicated longitudinal AKI-to-CKD patient cohort precluded direct assessment of the predictive value of serum lactate for AKI-to-CKD progression. Second, the effects of histone delactylation on renal fibrosis were investigated only in vitro; therefore, in vivo studies are needed to clarify the role of delactylation in renal injury and fibrogenesis. Third, renal fibrosis is a complex pathological process involving multiple cell types within the renal microenvironment, including fibroblasts, pericytes, and infiltrating immune cells. Both renal resident stromal cells and infiltrating immune cells may participate in and regulate lactate-mediated profibrotic events. The present study focused primarily on the TEC-autonomous mechanism of the lactate-Twist1 positive-feedback loop. Further studies are warranted to elucidate lactate-mediated crosstalk between tubular cells and other cellular populations in the fibrotic kidney.

**Fig. 7:**
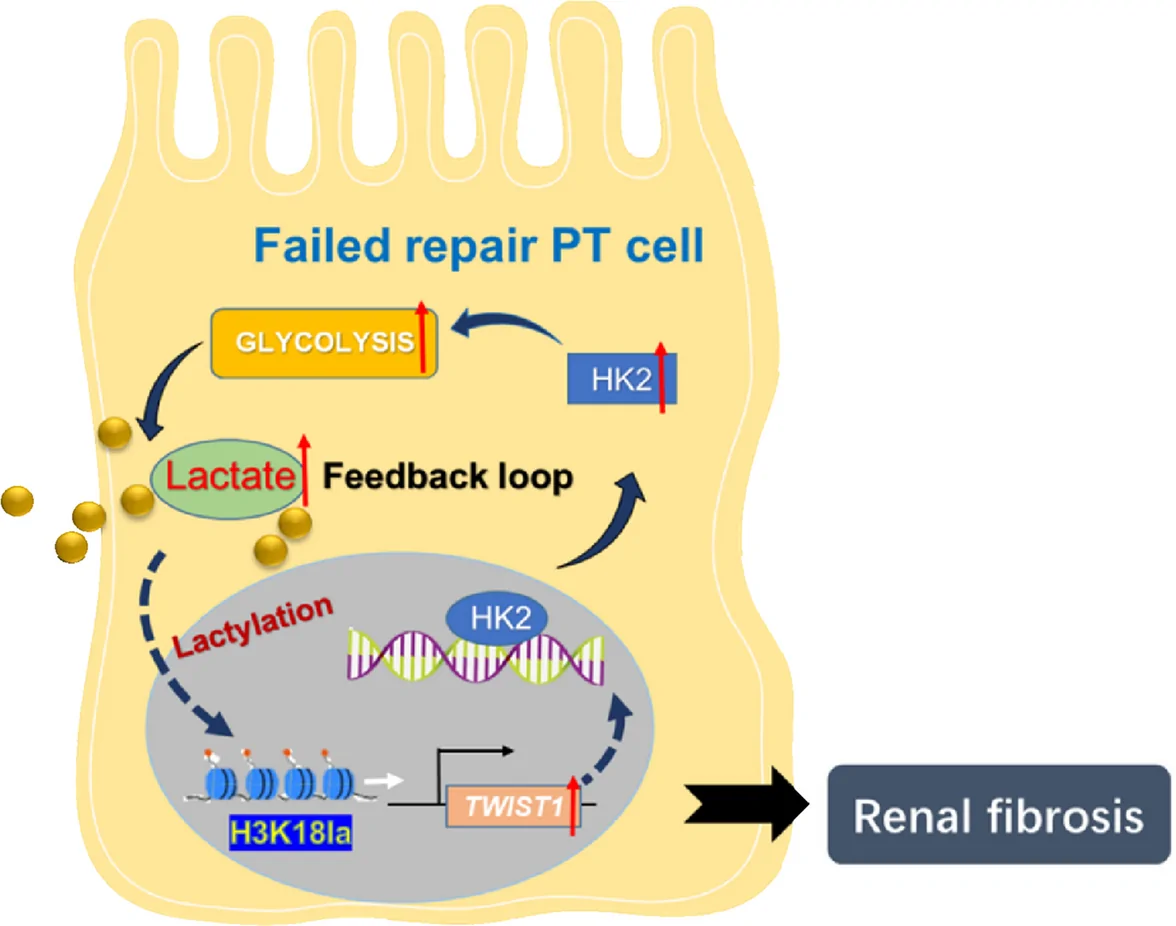
Schematic diagram of this research

A schematic illustration of the central mechanism identified in this study is presented in Fig. 7. Briefly, hypoxia-induced Twist1 promotes HK2-dependent glycolysis and lactate accumulation in TECs. Lactate subsequently induces H3K18 lactylation at the Twist1 promoter, further enhancing Twist1 transcription and establishing a self-amplifying metabolic-epigenetic feedback loop that drives tubular maladaptive repair and renal fibrosis. Nevertheless, systemic targeting of this glycolysis-centered axis may produce metabolic adverse effects, given the essential role of glycolysis in systemic energy homeostasis and the physiological functions of multiple normal tissues. These functions include basal energy supply in renal tubules, immune-cell activation, and normal tissue-repair processes. To minimize potential off-target effects, renal PT-specific delivery systems or selective modulators targeting the Twist1-HK2 regulatory axis, rather than broad-spectrum glycolysis inhibitors, may offer more feasible and safer strategies for clinical translation. Clinically, serum lactate may serve as both a prognostic biomarker and a therapeutic target. Disruption of this axis through glycolysis inhibition, modulation of lactylation, or Twist1 antagonism may provide a strategy to halt fibrosis and improve outcomes in CKD. Future studies should translate these mechanistic insights into cell-selective therapeutic approaches to address the unmet need for effective antifibrotic therapies in nephrology.

## Methods

### Ethical approval

The study protocol was reviewed and approved by the Ethics Committee of Xijing Hospital (Ethics ID: KY20223075-1). All animal procedures were approved by the Animal Experiment Administration Committee of the Fourth Military Medical University (Ethics ID: No. 20230366). Human data from the Medical Information Mart for Intensive Care IV (MIMIC-IV) v1.0 database were de-identified, and the study had no impact on clinical care. Consequently, the Ethics Committee waived the requirement for individual informed consent for the use of these data. Informed consent was obtained from all patients who provided renal biopsy specimens for this study.

### Kidney injury mouse models

Male C57BL/6 mice (8–10 weeks old; 22–25 g) and C57BL/6 J mice (8 weeks old; 25–30 g) were housed under standard conditions, with a 12-h light/dark cycle and ad libitum access to food and water. Animals were randomly assigned to the following groups: sham-operated controls (kidney separation only), unilateral ischemia–reperfusion injury (UIRI; right renal pedicle clamping at 37 °C for 30 min followed by reperfusion) [40–42], UIRI plus the glycolytic inhibitor 2-deoxy-D-glucose (2-DG), unilateral ureteral obstruction (UUO; left ureter ligation), and UUO plus 2-DG (≥ 12 mice per group). UIRI and UUO procedures were performed as previously described [40, 41]. To assess whether renal fibrosis in mice was mediated by lactate, separate cohorts received intraperitoneal injections of sodium lactate (1 g/kg) or saline for 3 or 14 days (≥ 5 mice per group). Kidneys were harvested 3 and 14 days after the procedure. Samples for single-nucleus RNA sequencing (snRNA-seq) were snap-frozen in liquid nitrogen following transcardial perfusion with PBS. All in vivo intervention experiments, including UIRI/UUO model establishment, 2-DG administration, and sodium lactate treatment, were conducted with strict randomization using a random-number table. No significant differences in baseline age, body weight, or general condition were observed among the experimental groups.

### Construction of proximal tubule-specific Twist1 gene knockout mice (PEPCK-Twist1(-/-))

PT-specific *Twist1* knockout mice (PT-*Twist1*^−/−^) were generated by crossing *Twist1*^flox/flox^ mice (Mutant Mouse and Research Center, University of North Carolina) with PEPCK-Cre mice provided by Prof. Dong Zheng (Xiangya Medical College). The progeny included the following genotypes: *Twist1*^flox/flox^/Cre (PT-*Twist1*^−/−^), *Twist1*^+/+^/Cre (Cre control), *Twist1*^flox/flox^ (Flox control), and *Twist1*^+/+^ (wild-type control). PT-*Twist1*^+/+^ littermates served as negative controls.

### Human kidney samples

Renal tissue samples were obtained from renal biopsies performed during routine clinical care at Xijing Hospital between 2020 and 2023. Samples from 23 patients with IgAN and paracancerous tissues from 13 patients serving as controls were formalin-fixed and paraffin-embedded (FFPE). All 4-μm sections were prepared from needle-core biopsy specimens collected during routine nephrology procedures.

### Immunohistochemistry and immunofluorescence staining of renal tissues

FFPE sections (4 μm) were deparaffinized in xylene and rehydrated through alcohol. Antigen retrieval was performed using 1 mM EDTA (pH 9.0) with steam heating. For immunohistochemistry, endogenous peroxidase activity was quenched with 3% H_2_O_2_ for 10 min. Non-specific binding was blocked with 5% normal goat serum (Vector Laboratories, S-1000) for 30 min at room temperature. Primary antibodies (Table S3) were applied in antibody diluent for 1 h at room temperature for immunohistochemistry or overnight at 4 °C for immunofluorescence.

For immunohistochemical analysis, sections were incubated with horseradish peroxidase (HRP)-conjugated secondary antibodies for 15 min, followed by diaminobenzidine development and hematoxylin counterstaining. For immunofluorescence, sections were incubated with Alexa Fluor 488-conjugated goat anti-rabbit IgG (1:500; A-11008, Thermo) and Alexa Fluor 594-conjugated goat anti-mouse IgG (1:500; A-11005, Thermo) for 2 h at room temperature.

### Histopathological staining of renal tissues

Renal tissues were fixed in 4% paraformaldehyde, paraffin-embedded, and sectioned at 2 μm for histological staining, including periodic acid–Schiff (PAS), hematoxylin and eosin (H&E), and Masson’s trichrome staining. Images were acquired using an Olympus BX53 microscope equipped with a DP27 digital camera. All histopathological evaluations, semiquantitative scoring of renal fibrosis, and quantitative analyses of immunohistochemical and immunofluorescence staining were performed independently by two researchers in a single-blinded manner. The investigators had no access to sample group assignments throughout the evaluation process.

### Clinical data source

The MIMIC-IV database was established in 2003 with funding from Beth Israel Deaconess Medical Center, the Massachusetts Institute of Technology, and the National Institutes of Health [43]. The database integrates multidisciplinary collaborations involving Massachusetts General Hospital, emergency and critical care clinicians, and computer scientists. As the largest open-access critical care database, it is derived from the intensive care unit (ICU) patient management system of Beth Israel Deaconess Medical Center. MIMIC-IV version 1.0 contains ICU admission data from 2008 to 2019. Serum lactate levels, in-hospital estimated glomerular filtration rate (eGFR), AKI severity grades, and mortality outcomes, including ICU and in-hospital mortality, were extracted for all patients admitted to the ICU.

### Mendelian randomization (MR)

Genetic instruments for CKD and metabolites were obtained from the FinnGen consortium (finn-b-N14_CHRONKIDNEYDIS) and metabolome-wide association studies. Metabolite quantification data (GCST90199621–GCST90201020) were obtained from Chen et al. [39] (total sample size, n = 7,814) and covered 1,459 metabolites. Following linkage disequilibrium clumping (r^2^ < 0.001; 10-Mb window), 2,545,582 genome-wide significant single-nucleotide polymorphisms (*P* < 5 × 10^–6^) were retained. Bidirectional two-sample MR analysis was performed using the Two SampleMR package [44] to evaluate the causal effect of serum lactate on CKD risk (forward analysis) and the reverse causal effect of CKD on serum lactate levels (reverse analysis), with rigorous harmonization of effect alleles. Primary causal estimates were derived using inverse-variance weighted random-effects regression [45], with MR-Egger [46] and weighted-mode [47] methods used as complementary approaches. Robustness was evaluated by: (1) Cochran’s Q statistic for heterogeneity assessment [44]; (2) MR-Egger intercept testing for directional pleiotropy [46]; (3) MR-PRESSO outlier correction when the Global Test *P* value was < 0.05 [48]; and (4) leave-one-out sensitivity analyses.

### 10 × genomics-based snRNA-seq and data processing

snRNA-seq was performed using the 10 × Genomics platform with Cell Ranger v6.1.2 [49]. Barcoded gel bead-in-emulsions (GEMs) were generated through microfluidic encapsulation, followed by cDNA library construction via reverse transcription and sample indexing [50]. Viable cells, with viability exceeding 90% as assessed by Trypan blue staining, were processed by OE Biotech (Shanghai, China) according to the manufacturer’s protocols. Data were analyzed using Seurat v4.0.5 [51]. Cells were filtered according to the following criteria: 200–7,000 detected genes and < 5% mitochondrial gene content. LogNormalize normalization was performed before selection of highly variable genes (2,000 genes using the “vst” method), principal component analysis (PCA)-based dimensionality reduction, and batch correction using Harmony v0.1.0 [52]. Clustering was performed at a resolution of 0.6, and marker genes were identified using the Wilcoxon test (adjusted *P* < 0.05 and |logFC|> 0.25), enabling manual cell-type annotation. Pseudotime trajectory analysis of PT subtypes was conducted using Monocle v2.18.0 [53] based on variable genes, followed by branch-specific gene clustering. Pathway enrichment analyses were performed using WebGestalt [54] and KOBAS [55] with a hypergeometric test threshold of *P* < 0.001. Gene set variation analysis (GSVA) scores were calculated using an R package [56] to quantify pathway activity across clusters.

### RNA-Seq and data analysis

Total RNA was extracted from mouse kidneys using the Direct-zol RNA MicroPrep Kit (Zymo Research, R2060), and RNA quality was assessed using an Agilent TapeStation 4200. Libraries were prepared using the Illumina TruSeq Stranded mRNA Kit and subjected to paired-end 150-bp sequencing on a NovaSeq 6000 platform. Reads were aligned to the UCSC mm10 reference genome using Kallisto (v0.50), followed by differential expression analysis with edgeR. Human CKD RNA-sequencing data were obtained from the GEO database (GSE66494) [57].

### Cell culture

HK-2 cells (ATCC CRL-2190) were cultured under normoxic conditions (5% CO_2_/20% O_2_) [58] or hypoxic conditions (1% O_2_/5% CO_2_/95% N_2_) using an established in vitro model that simulates AKI-to-CKD progression [59].

### Western blotting

Proteins were extracted from mouse kidneys using RIPA buffer supplemented with protease inhibitors, as previously described [60]. Protein concentrations were determined using a BCA assay kit (Thermo Scientific, 23,225), and 30 μg of protein per lane was loaded for SDS-PAGE. Membranes were incubated with primary antibodies (Table S3) for 12 h at 4 °C, followed by HRP-conjugated secondary antibodies for 2 h at room temperature. Experiments were performed in triplicate.

### Quantitative Real-Time PCR (qRT-PCR)

RNA was extracted from cells and kidney tissues using RNAiso Reagent (Takara Bio). Quantitative reverse-transcription PCR (qRT-PCR) was performed using a Roche LC480 system and SYBR Green Master Mix (Vazyme). Relative gene expression was calculated using the 2^−ΔΔCt^ method. Primer sequences are provided in Table S4.

### Untargeted metabolomics and measurement of metabolites

LC–MS/MS analysis was performed using an UltiMate 3000 HPLC system (Thermo, USA) equipped with an ACQUITY UPLC HSS T3 column (150 × 2.1 mm, 1.8 μm; Waters), maintained at 40 °C with a flow rate of 0.25 mL/min. For positive-ion mode, the mobile phases were 0.1% formic acid in water (A) and acetonitrile (B). For negative-ion mode, the mobile phases were 5 mM ammonium formate in water (A) and acetonitrile (B). Raw data were converted to mzXML format. Peak detection and normalization were performed using the XCMS package in R (v4.0.3). Metabolites were identified by comparison with the METLIN, MoNA, HMDB, and BioDeep (BioNovoGene, Suzhou, China) databases. Normalized data matrices were imported into SIMCA 14.1. Unsupervised PCA was used to assess overall sample separation, followed by supervised partial least-squares discriminant analysis (PLS-DA) and orthogonal partial least-squares discriminant analysis (OPLS-DA). Permutation testing was performed to validate the supervised models and prevent overfitting. Cultured cells were homogenized in lysis buffer by ice-cold sonication (300 W; 3-s on/7-s off cycles for 3 min), followed by centrifugation at 12,000 × g for 10 min at 4 °C. Lactate concentrations in the supernatants were measured using a Lactate Colorimetric Assay Kit (Solarbio, BC2235) according to the manufacturer’s instructions.

### Oxamic acid (OA), 2-DG, and A485 treatment

For in vivo experiments, 2-DG was administered at 150 mg/kg/day [7], with saline serving as the vehicle control. For in vitro experiments, HK-2 cells were treated with 2-DG (5 mM; Sigma-Aldrich, D8375), oxamic acid (5 μM; MCE, HY-W013032A), and A485, a potent p300/CBP inhibitor (10 μM; MCE, HY-107455).

### Hexokinase 2, lactate dehydrogenase A, and pyruvate kinase isozyme activity and lactate measurement

The enzymatic activities of HK2, LDHA, and PKM2 were assessed in cell lysates prepared using the corresponding assay buffers. Activities were quantified using colorimetric assay kits according to the manufacturers’ protocols (HK2, A077-3–1; LDHA, A076-1–1; PKM2, A020-1–1; Jiancheng Bioengineering, Nanjing, China).

### Histone extraction

Histones were extracted by acid extraction. Cells or tissues were lysed in buffer containing 10 mM Tris–HCl, 1 mM KCl, 1.5 mM MgCl_2_, and 1 mM DTT (pH 8.0), supplemented with protease inhibitors. Nuclei were subjected to acid extraction in 0.2 M H_2_SO_4_ overnight at 4 °C, followed by centrifugation at 16,000 g for 10 min at 4 °C. Supernatants were precipitated with trichloroacetic acid to a final concentration of 35%. The histone pellets were washed, dried, resuspended in H_2_O, and subjected to Western blotting analysis.

### Plasmids construction and transfection

*TWIST1* expression vectors and small interfering RNAs (siRNAs) were constructed according to established protocols. *LDHA* cDNA derived from hypoxia-treated HK-2 cells was subcloned into pcDNA3.1, whereas *LDHA*-targeting siRNA sequences were inserted into pSilencer3.1U6neo (Genecem). Scrambled sequences in pSilencer3.1 and empty pcDNA3.1 vectors were used as controls. H3K18 mutant plasmids were generated by Genecarer (Xi’an, China). For transfection, HK-2 cells seeded in 12-well plates were transfected using Lipofectamine 2000 (Invitrogen, 11,668,019) with 40 pM siRNA pools targeting *LDHA*, *TWIST1*, or negative control sequences, or with cDNA vectors encoding *LDHA* or H3K18. The culture medium was replaced after 6 h.

### Luciferase reporter assay

Luciferase reporter assays (Promega) were performed in 24-well plates when cells reached 50%–70% confluence. Cells were co-transfected with promoter constructs, including empty vector, Twist1, or Twist1 mutant constructs, and β-galactosidase (0.1 mg) as an internal control. After incubation for 24 h, cell lysates were analyzed for luciferase activity. Blank-subtracted luciferase activity was normalized to that of the empty-vector control.

### Chromatin immunoprecipitation-qPCR (ChIP)

Chromatin immunoprecipitation was performed using a Millipore ChIP Kit with formaldehyde-fixed HK-2 cells. Cross-linked chromatin was sonicated and immunoprecipitated overnight at 4 °C using H3K18lac and Twist1 antibodies coupled to Protein G magnetic beads. The immunoprecipitated chromatin was subsequently purified and de-cross-linked for RT-PCR and bulk sequencing analyses.

### Statistical analysis

Data are presented as the mean ± SEM. Statistical analyses were performed using GraphPad Prism 8.0, R, and Stata. Two-tailed unpaired t-tests or one-way/two-way ANOVA followed by Tukey’s post hoc test were used as appropriate. Statistical significance was defined as *P* < 0.05 or *P* < 0.01. Specifically, *P* values for comparisons between two groups were calculated using two-tailed unpaired t-tests, whereas *P* values for comparisons among multiple groups were derived from one-way or two-way ANOVA followed by Tukey’s post hoc test.

Using MIMIC data, serum lactate levels at admission were compared across AKI stages. Scatterplots were constructed to illustrate the correlation between serum lactate levels and eGFR, stratified by AKI severity, and smooth curve fitting was performed to quantify the association between lactate levels and mortality among ICU admissions. In addition, generalized additive models were used to analyze AKI prevalence and variations in serum lactate levels, whereas threshold-effect analysis was conducted to identify critical lactate thresholds associated with increasing mortality.

## Supplementary Information

Supplementary Material 1.

## Data Availability

The raw data supporting the conclusions of this article will be made available by the authors without undue reservation. Data generated and analyzed in the current study are available from CRA047100 and CRA047101 in China National Center for Bioinformation. Publicly available datasets can be accessed through the Gene Expression Omnibus (GEO) database: GSE66494, https://www.ncbi.nlm.nih.gov/geo/query/acc.cgi?acc=GSE66494; and GSE60685, https://www.ncbi.nlm.nih.gov/geo/query/acc.cgi?acc=GSE60685. The code used in this study is available from the corresponding author upon reasonable request. Genome-wide association study summary statistics from the IEU GWAS database may be accessed by submitting requests to the relevant data custodians. The analytical scripts used for Mendelian randomization analysis are also available through the GitHub repository for the “TwoSampleMR” R package: https://github.com/MRCIEU/TwoSampleMR/.
