# Supplementary material for: Twist Family BHLH Transcription Factor 1 orchestrates hypoxia-lactate crosstalk to bridge glycolytic flux and tubular maladaptation in fibrotic kidneys
Source: Mol Biomed. 2026 Aug 24;7:149. doi: 10.1186/s43556-026-00555-9 (PMC13504060; doi:10.1186/s43556-026-00555-9)
Supplement: Supplementary file 1 [file 43556_2026_555_MOESM1_ESM.pdf]

Twist Family BHLH Transcription Factor 1 Orchestrates Hypoxia-Lactate Crosstalk to Bridge Glycolytic Flux and Tubular Maladaptation in Fibrotic Kidneys

Authors: Zhixiang Yu[1,3†], Yuzhan Zhang[1†], Xiao Bai[1†], Jin Zhao[1], Lei Wei[1], Jingli Gao[1], Hao Wu[1], Rui Ma[1,2], Xiaoxuan Ning[1,2*], Shiren Sun[1*]

Affiliations:

[1]Department of Nephrology, Xijing Hospital, Fourth Military Medical University; Xi'an, China.

[2]Department of Geriatrics, Xijing Hospital, Fourth Military Medical University; Xi'an, China.

[3]Department of Nephrology, The Ninth Medical Center of Chinese PLA General Hospital, Beijing, China

*Corresponding authors. Shiren Sun, MD., PhD. Email: sunsrsun@163.com and sunshirenamedmail.com.cn

Xiaoxuan Ning MD., PhD Email: ningxx01@fmmu.edu.cn

[†]These authors contribute the same to this manuscript.

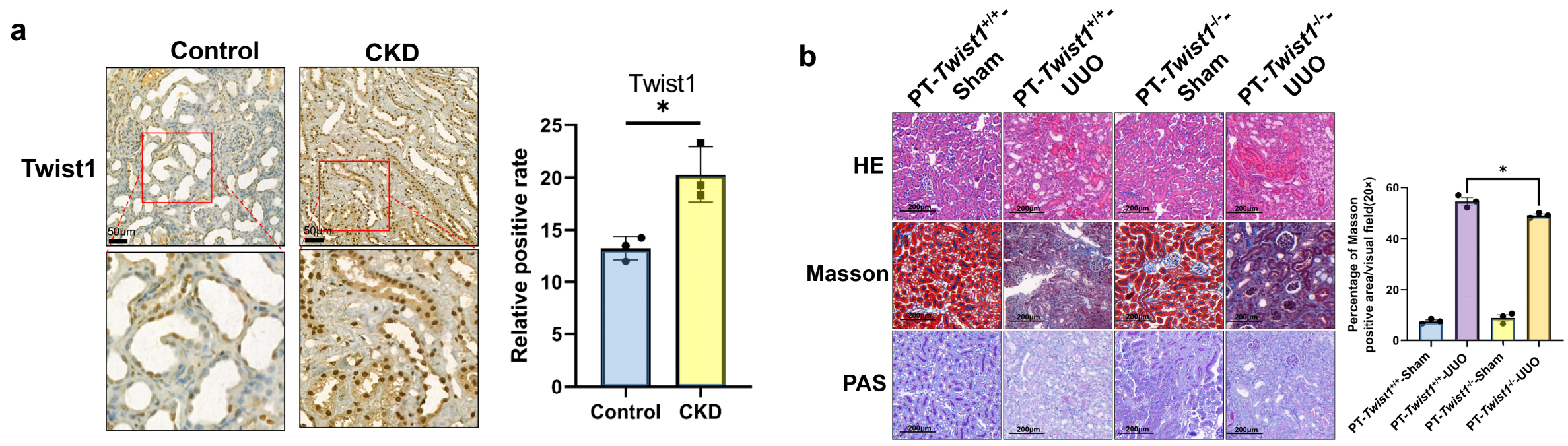

Figure S1 Twist1 expression in human CKD kidneys and its profibrotic role in mouse UUO and UIRI models. **(a)** Immunohistochemical staining of Twist1 in para-cancerous renal tissue and renal tissue of patients with CKD, the magnification is 20 ×, the red box is the local magnified image, statistical chart of the positive rate of immunohistochemical staining; *$P < 0.05$, n = 3. **(b)** HE, Masson and PAS staining methods were used to detect the morphology and degree of fibrosis in the kidneys of control mice (sham) and UUO mice. The magnification was 20 ×. Statistical analysis of collagen area, comparing the *Twist1* knockout (PT-*Twist1*$^{-/-}$) UUO group with the transgenic (PT-*Twist1*$^{+/+}$) UUO group, *$P < 0.05$, n = 3 mice per group.

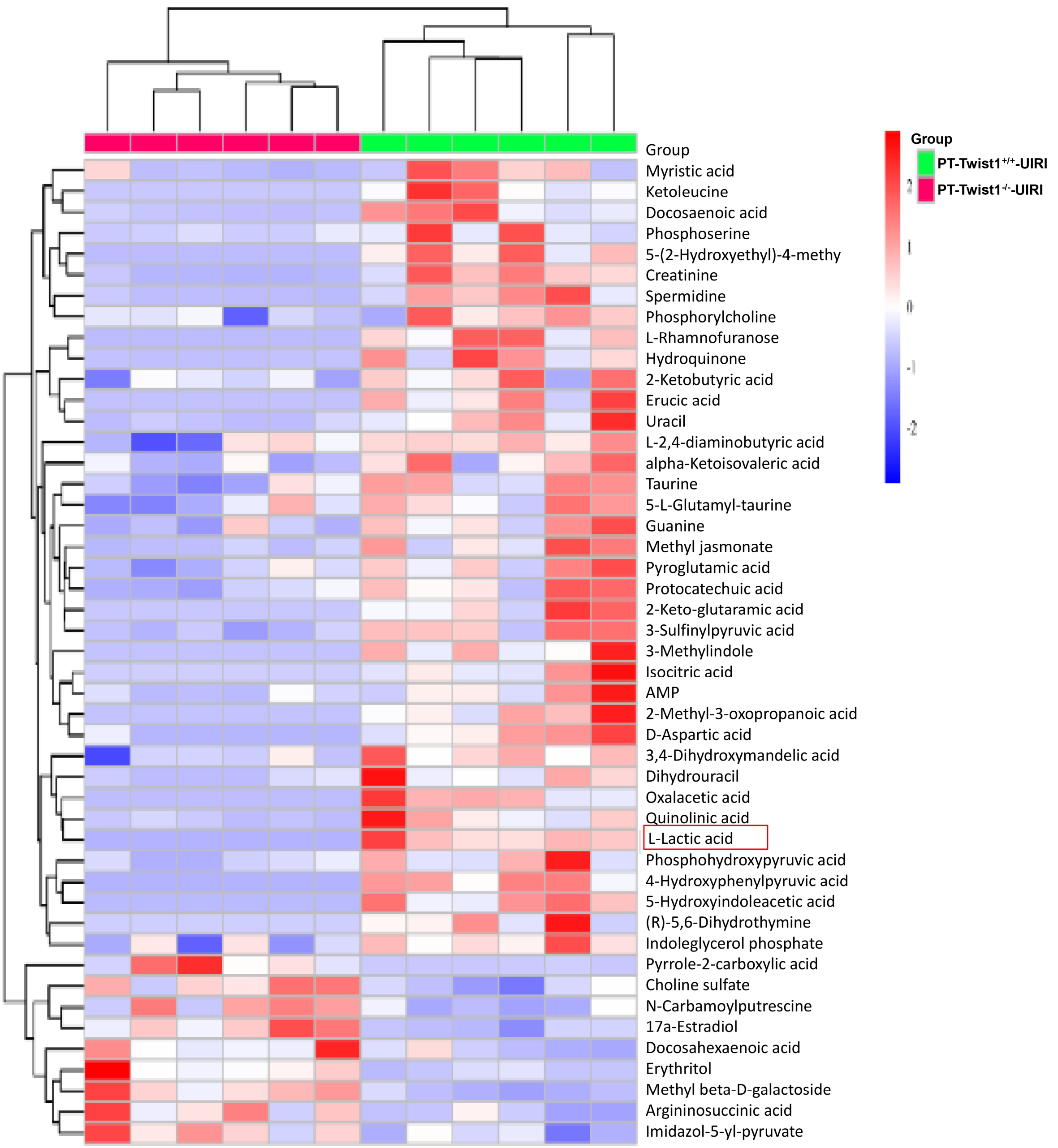

Figure S2. Heatmap of differentially abundant metabolic intermediates in whole-kidney tissues from PT-Twist1$^{-/-}$ and PT-Twist1$^{+/+}$ UIRI mice. Lactate abundance in kidney tissues from the two groups of mice is shown. $^{**}P < 0.01$, n = 6 mice per group.

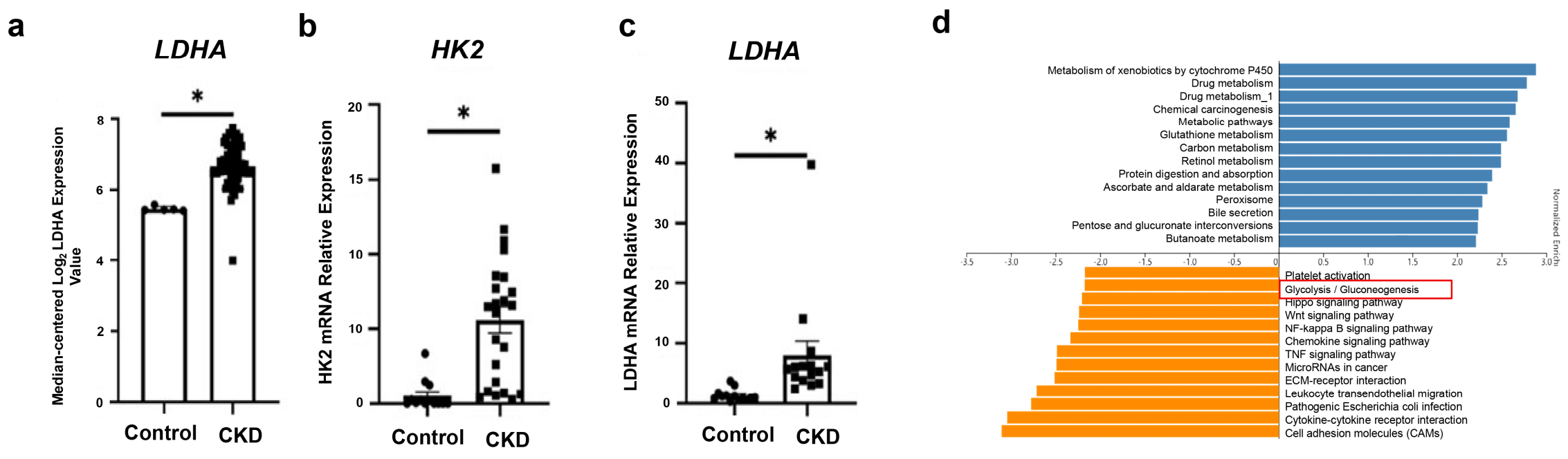

Figure S3. Glycolytic genes *HK2* and *LDHA* are upregulated in human CKD and IgAN kidneys with pathway enrichment analysis. (a) Comparison of LDHA mRNA expression in kidney tissues from 48 patients with CKD and 5 healthy controls using the Nephroseq database. *$P < 0.05$, n = 53. (b, c) *HK2* **(b)** and *LDHA* **(c)** mRNA expression levels were also increased in kidney biopsies from patients with CKD caused by IgA nephropathy (IgAN; n = 23) compared with controls (n = 13). *$P < 0.05$. **(d)** Pathway enrichment analysis of kidney tissues from 23 patients with IgAN-associated CKD compared with 13 controls. Orange bars indicate pathways enriched in CKD kidneys, whereas blue bars indicate pathways enriched in normal kidneys. Bar length represents the enrichment score, with larger absolute values indicating stronger pathway enrichment. All listed pathways reached statistical significance ($P < 0.05$).

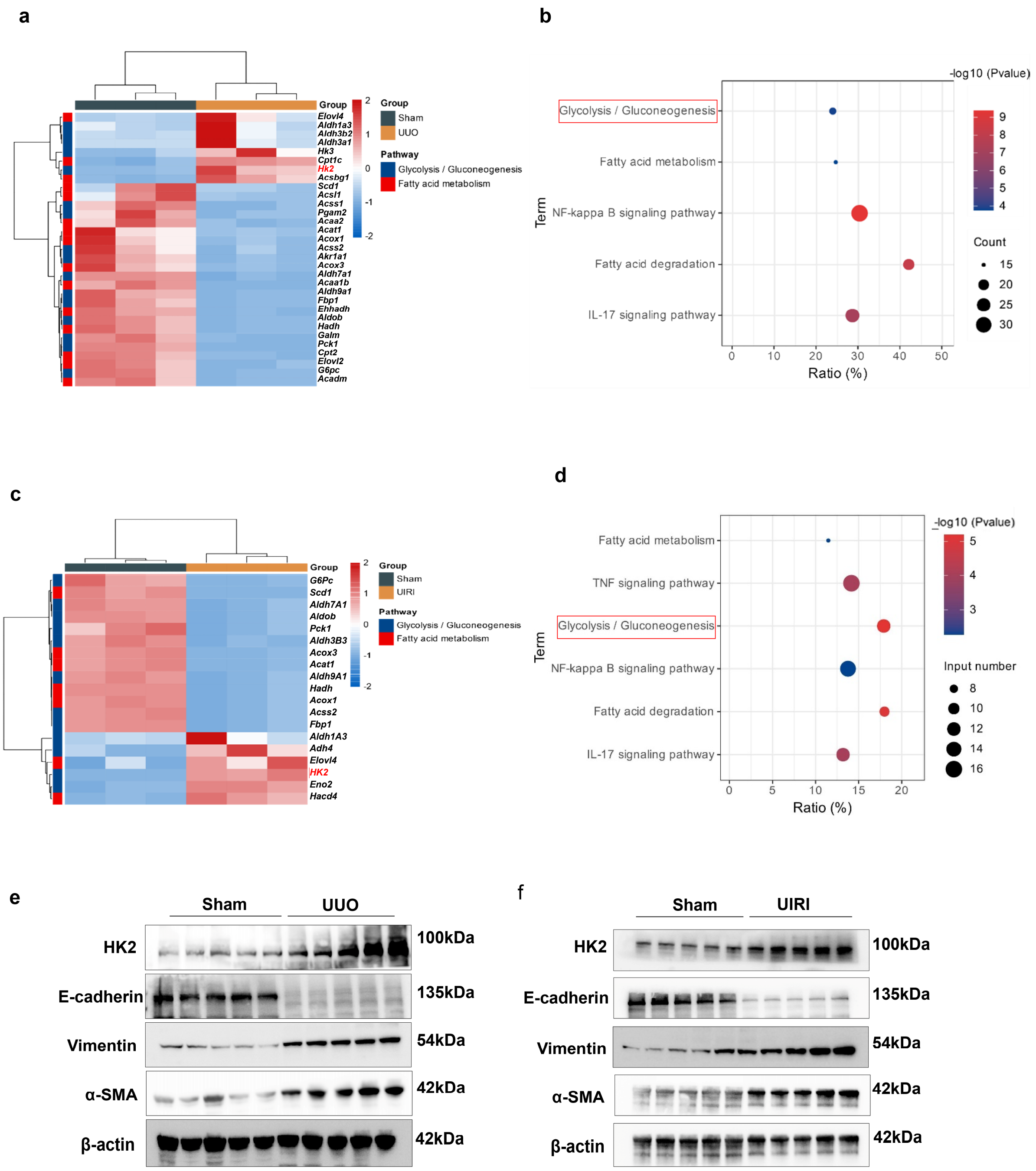

Figure S4. Renal glycolysis and fibrosis are altered in UUO and UIRI mice. (a) Heatmap of genes involved in the glycolytic pathway in UUO mice (n = 3 per group). **(b)** Bubble plot showing KEGG pathway enrichment of differentially expressed genes in kidneys from UUO mice. **(c)** Heatmap of genes involved in the glycolytic pathway in UIRI mice (n = 3 per group). **(d)** Bubble plot showing KEGG pathway enrichment of differentially expressed genes in kidneys from UIRI mice. Bubble size and color represent the number of enriched genes and the enrichment *P* value, respectively. **(e)** Western blotting analysis of Vimentin,

HK2, α-SMA, and E-cadherin expression in whole kidneys from UUO mice (n = 5 per group). **(f)** Western blotting analysis of Vimentin, HK2, α-SMA, and E-cadherin expression in whole kidneys from UIRI mice (n = 5 per group).

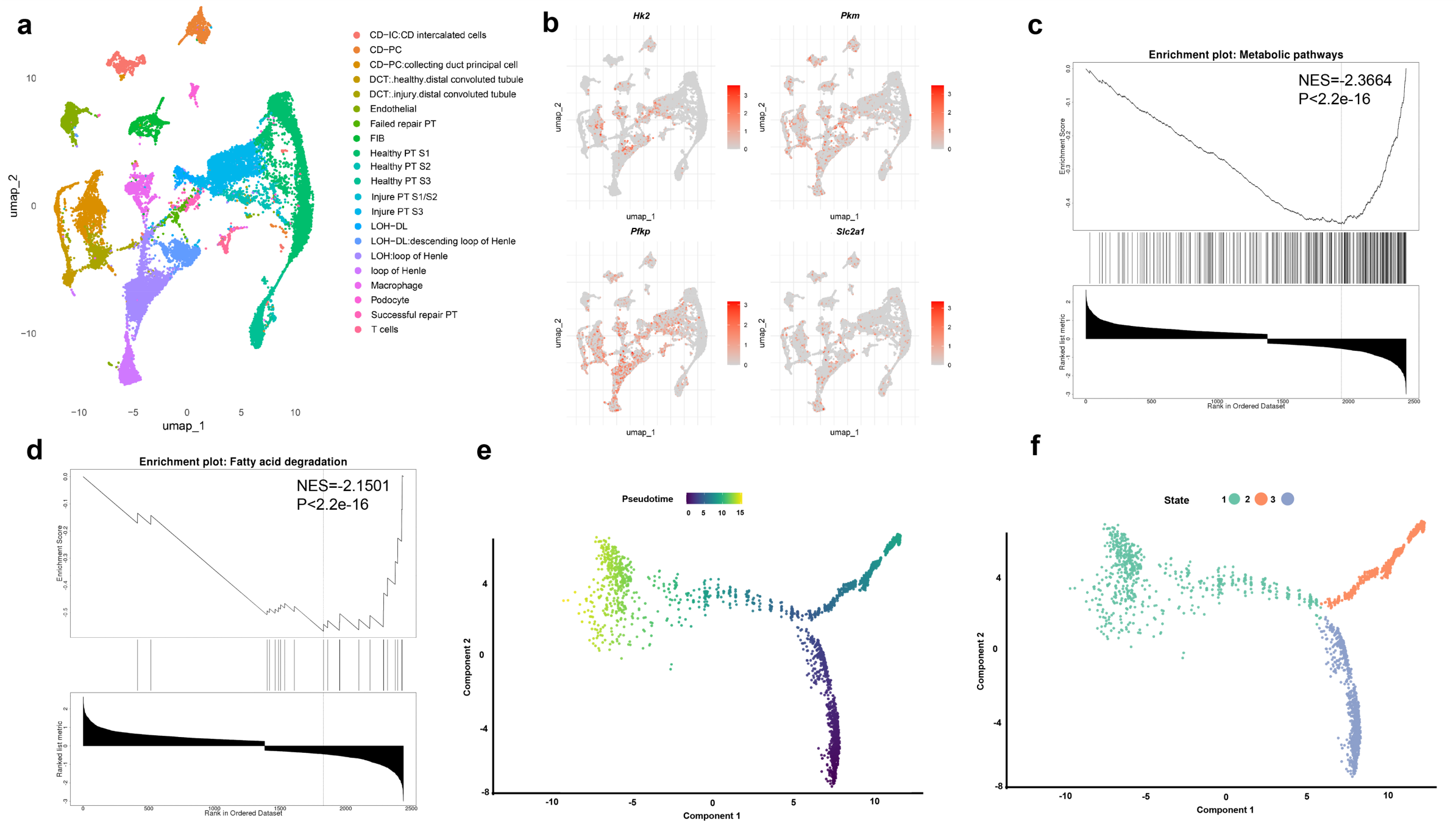

Figure S5. Single-cell transcriptomic analysis reveals glycolysis activation and cell trajectory dynamics in the mouse kidney. (a) UMAP visualization of mouse kidney cells. Different colors represent distinct cell types. **(b)** UMAP plots showing the expression of glycolysis-associated genes. **(c)** Gene set enrichment analysis showing enrichment of differentially expressed genes in metabolic pathways (NES = −2.3664, $P < 2.2 \times$ 10flox/flox−16) and **(d)** fatty acid degradation (NES = −2.1501, $P < 2.2 \times$ 10flox/flox−16). **(e)** Pseudotime trajectory analysis of kidney cells. Color intensity indicates progression from early to late pseudotime. **(f)** Trajectory analysis of cell states, with different colors representing distinct differentiation states.

Abbreviations: CD-PC, principal cells of the collecting duct; DCT, distal convoluted tubule; Pod, podocytes; LOH-DL, descending limb of the loop of Henle; Endo, endothelial cells; Act Fib 1, activated fibroblasts 1; Act Fib 2, activated fibroblasts 2; CD-IC, intercalated cells of the collecting duct; Macro, macrophages; LOH-AL, ascending limb of the loop of Henle; URO, urothelium; PT, proximal tubule; Novel, undefined novel cell subtype.

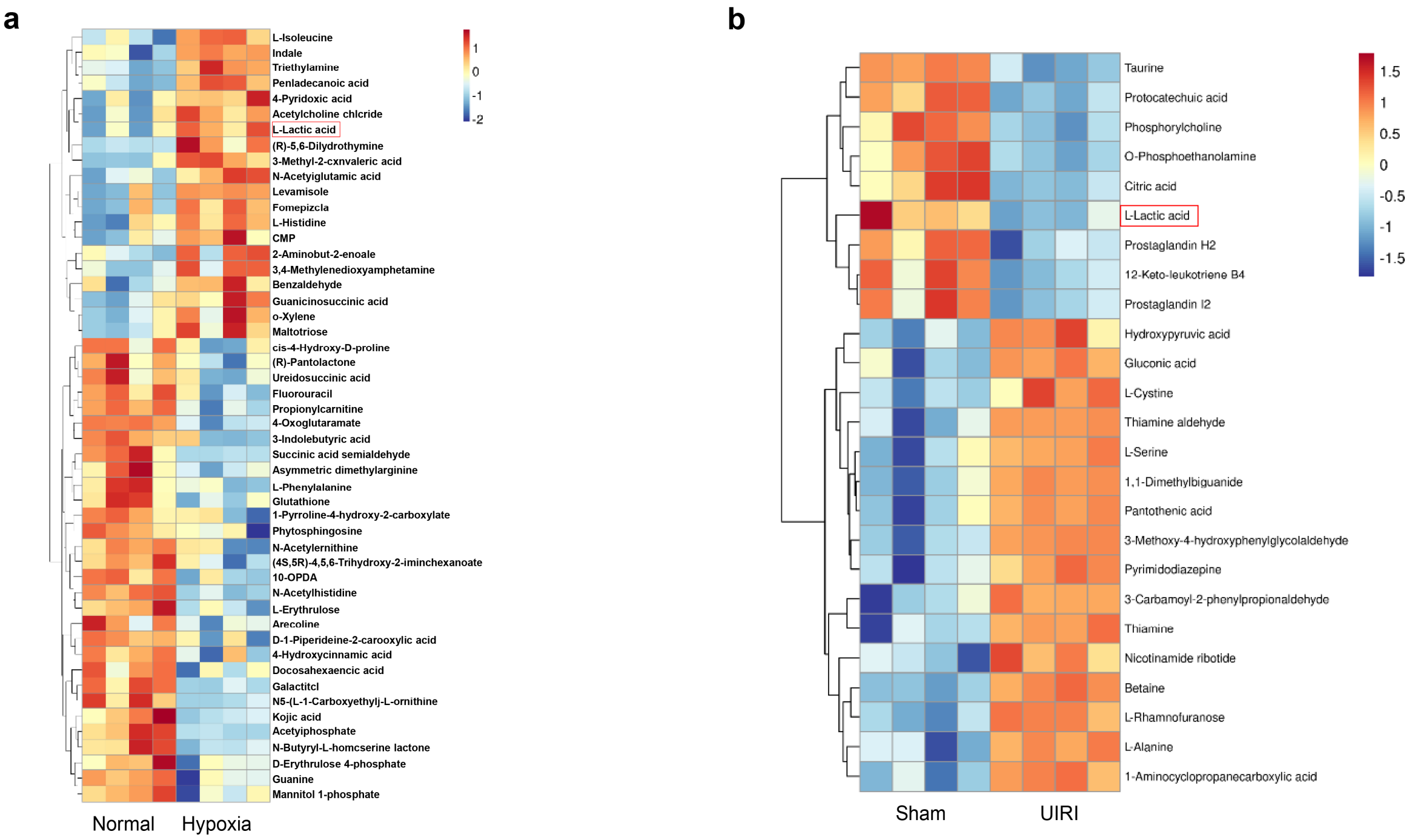

Figure S6. Lactate accumulation occurs under hypoxic-ischemic conditions both *in vivo* and *in vitro*. (a) Complete heatmap of differentially abundant metabolites identified by metabolomic analysis in HK-2 cells cultured under hypoxic and normoxic conditions (n = 3 per group). **(b)** Complete heatmap of differentially abundant metabolic intermediates in kidney tissues from sham and UIRI mice (n = 4 per group).

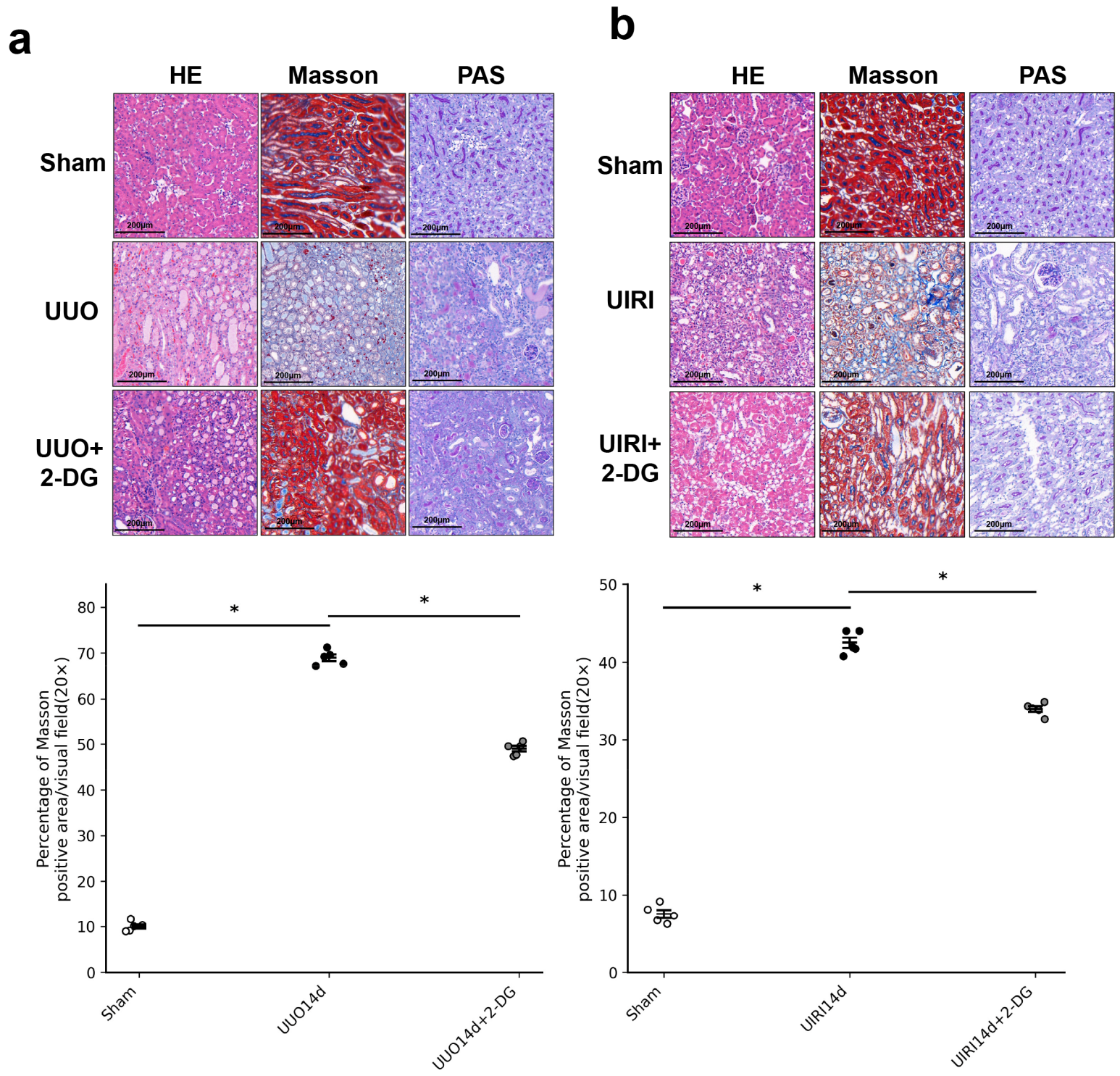

Figure S7. 2-DG ameliorates renal fibrosis in mouse UUO and UIRI models. (a) H&E, Masson, and PAS staining were used to assess renal morphology and fibrosis in kidneys from control, UUO, and UUO + 2-DG mice. Magnification, × 20. $P < 0.05$, n = 3. **(b)** H&E, Masson, and PAS staining were used to assess renal morphology and fibrosis in kidneys from control, Day 14 UIRI, and Day 14 UIRI + 2-DG mice. Magnification, × 20. $P < 0.05$, n = 3.

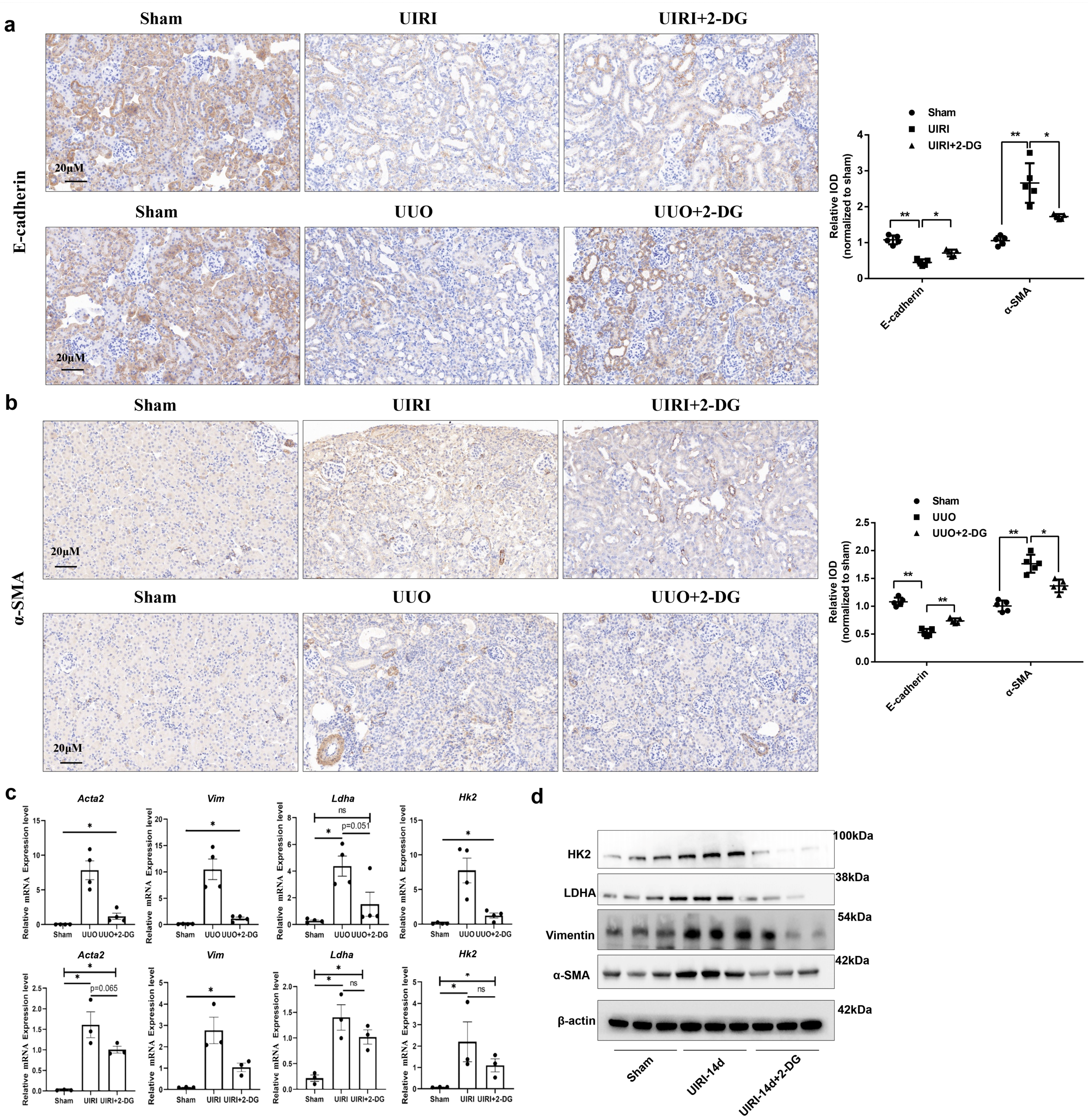

Figure S8. Anti-fibrotic effects of 2-DG in mouse models of renal fibrosis.

(a, b) Immunohistochemical staining of E-cadherin **(a)** and α-SMA **(b)** in kidney tissues from sham, UIRI, UUO, UIRI + 2-DG, and UUO + 2-DG mice at Day 14. Magnification, × 20. $P < 0.05$, $P < 0.01$. **(c)** mRNA expression of *Acta2*, *Vim*, *Ldha*, and *Hk2* in the UUO and UIRI mouse models. $P < 0.05$, n = 4 per UUO group and n = 3 per UIRI group. **(d)** Western blotting analysis of HK2, LDHA, Vimentin, and α-SMA in kidneys from UIRI mice with or without intraperitoneal 2-DG treatment (n = 3 per group).

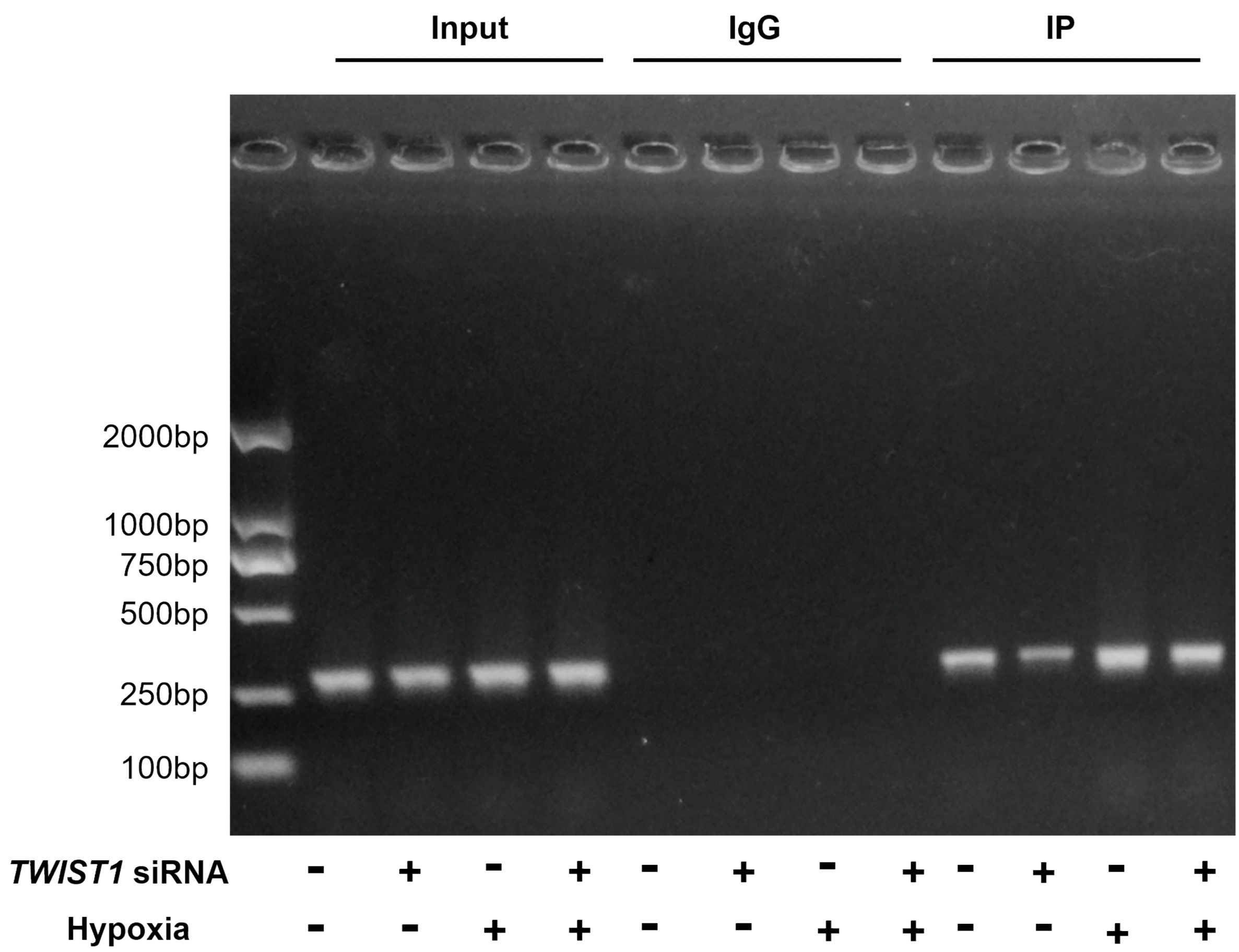

Figure S9. ChIP assay demonstrates *TWIST1* binding to the *HK2* promoter under hypoxic conditions.

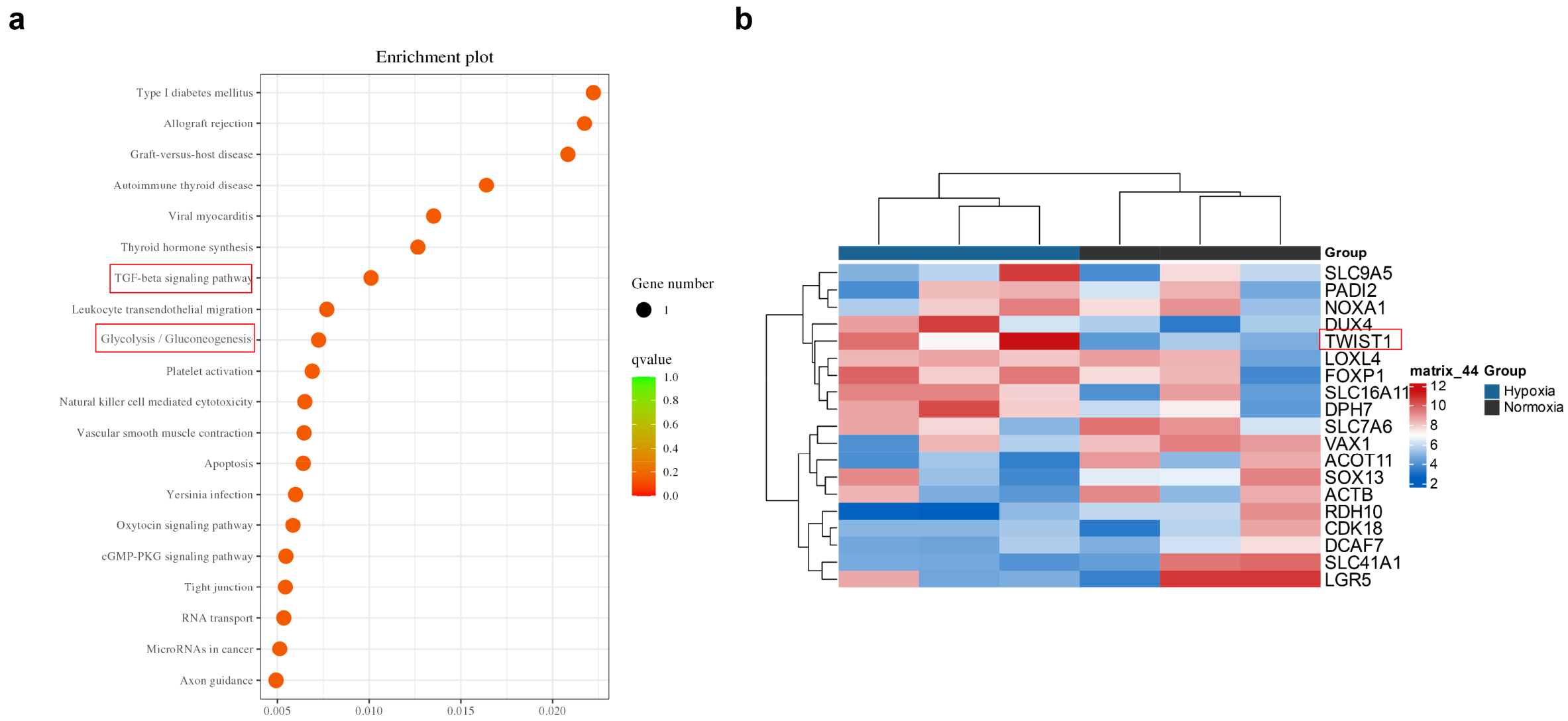

Figure S10. H3K18lac downstream transcription factors in hypoxic HK-2 cells. (a) KEGG pathway enrichment analysis of downstream transcription factors associated with H3K18lac that were significantly altered between hypoxic and normoxic HK-2 cells. **(b)** Heatmap showing the expression of H3K18lac-associated downstream transcription factors that were significantly altered between hypoxic and normoxic HK-2 cells (n = 3 per group).

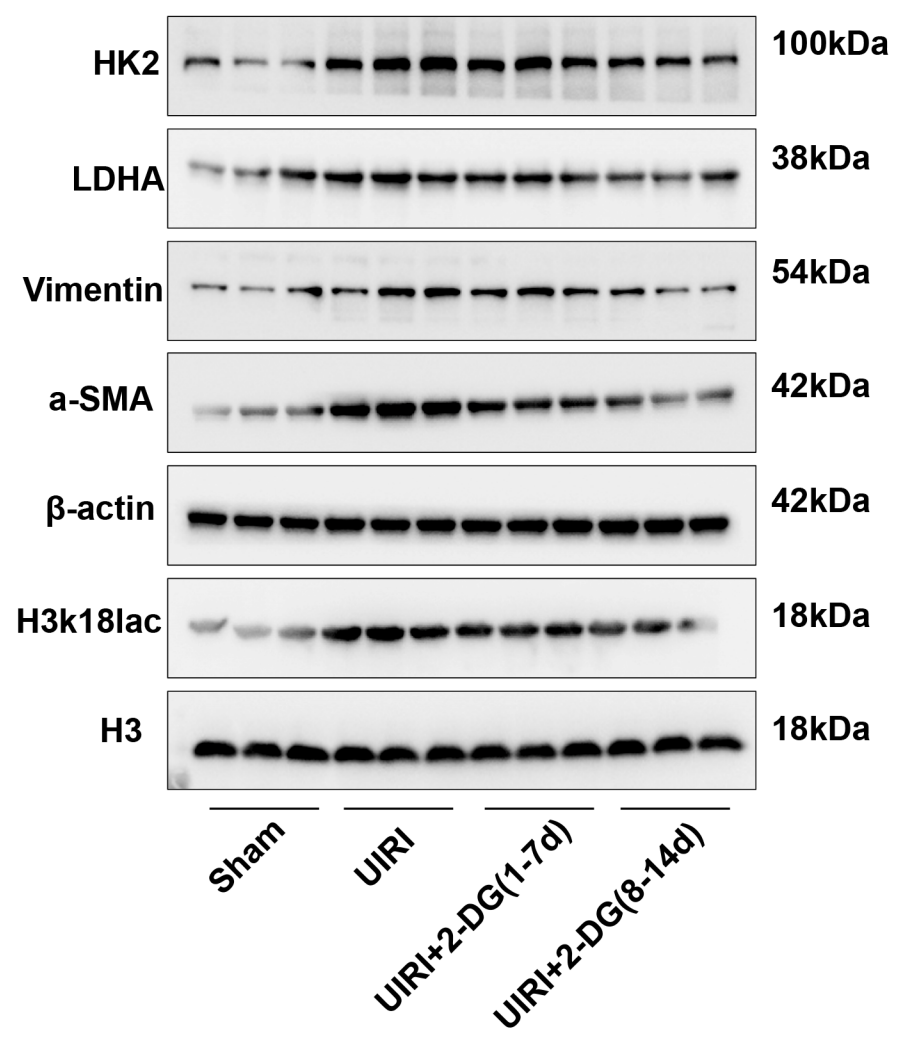

Figure S11. Western blotting analysis of glycolytic enzymes (HK2 and LDHA), fibrotic markers (Vimentin and α-SMA), and histone lactylation (H3K18lac) in kidney tissues from sham-operated mice, UIRI mice, UIRI mice treated with 2-DG from days 1–7, and UIRI mice treated with 2-DG from days 8–14.

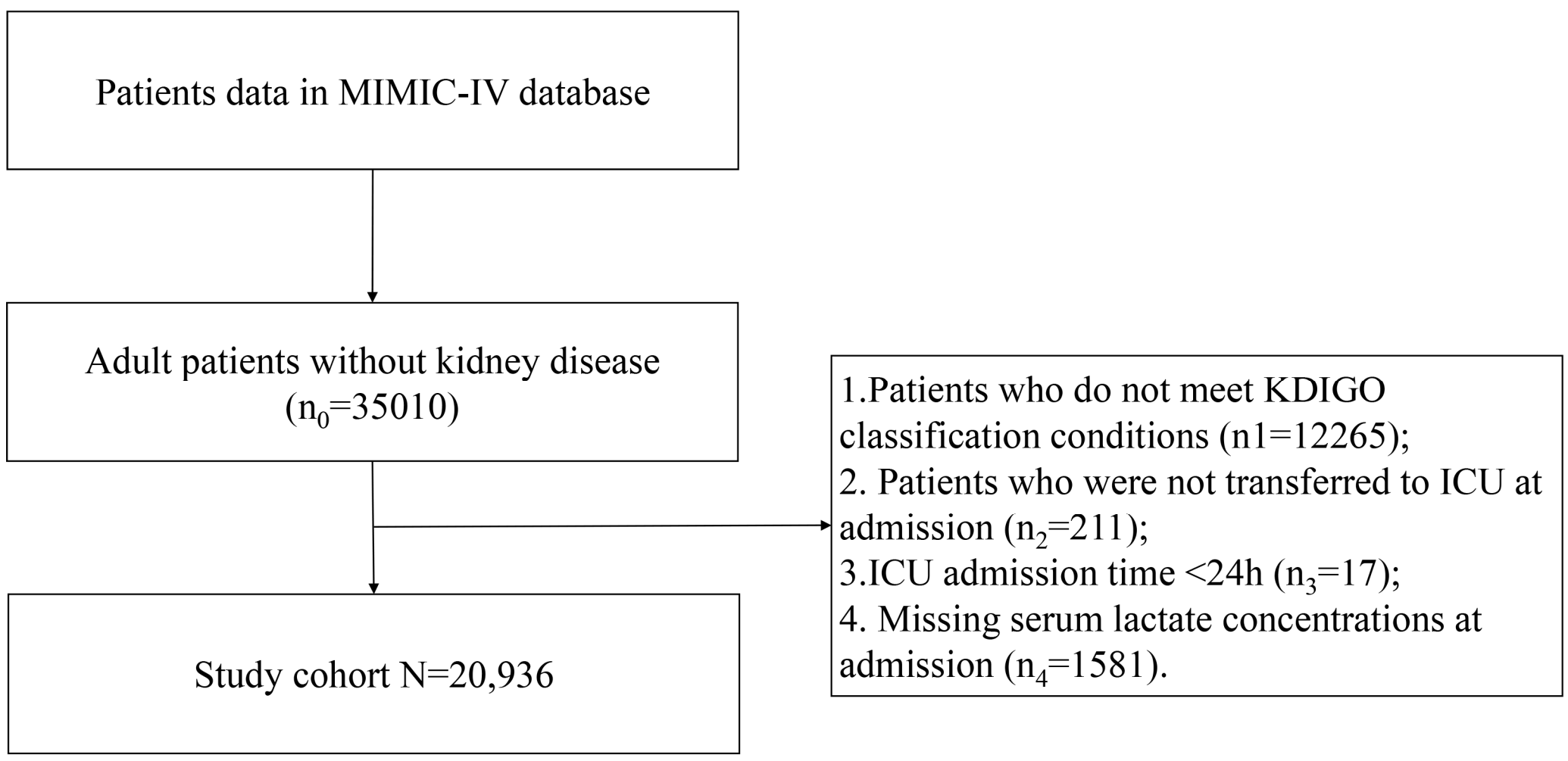

Figure S12. Flowchart of patient screening and enrollment from the MIMIC-IV database.

Exposure	nsnp	method	pval	OR(95% CI)
Propionylglycine	7	Weighted median	**<0.001**	1.286 (1.166 to 1.419)
	7	Inverse variance weighted	**<0.001**	1.229 (1.092 to 1.382)
	7	Weighted mode	**0.002**	1.301 (1.178 to 1.438)
3-hydroxypyridine sulfate	14	Weighted median	**<0.001**	1.214 (1.130 to 1.303)
	14	Inverse variance weighted	**<0.001**	1.180 (1.110 to 1.254)
	14	Weighted mode	**<0.001**	1.213 (1.128 to 1.304)
Gamma-glutamylglycine	13	Weighted median	**0.011**	0.809 (0.687 to 0.953)
	13	Inverse variance weighted	**0.002**	0.825 (0.732 to 0.930)
	13	Weighted mode	0.059	0.744 (0.564 to 0.983)
N-carbamoylalanine	8	Weighted median	**0.009**	0.767 (0.629 to 0.935)
	8	Inverse variance weighted	**<0.001**	0.765 (0.656 to 0.892)
	8	Weighted mode	0.055	0.709 (0.530 to 0.950)
2-ketocaprylate	10	Weighted median	**<0.001**	1.233 (1.092 to 1.391)
	10	Inverse variance weighted	**0.002**	1.176 (1.062 to 1.303)
	10	Weighted mode	**0.005**	1.252 (1.109 to 1.413)
N-acetyl-2-aminoadipate	13	Weighted median	**<0.001**	0.850 (0.775 to 0.933)
	13	Inverse variance weighted	**<0.001**	0.874 (0.811 to 0.941)
	13	Weighted mode	**0.011**	0.857 (0.775 to 0.947)
Creatine	12	Weighted median	**<0.001**	1.371 (1.154 to 1.629)
	12	Inverse variance weighted	**0.001**	1.415 (1.145 to 1.748)
	12	Weighted mode	**<0.001**	1.537 (1.279 to 1.847)
Glycine	12	Weighted median	**<0.001**	1.179 (1.109 to 1.254)
	12	Inverse variance weighted	**<0.001**	1.148 (1.076 to 1.225)
	12	Weighted mode	**<0.001**	1.181 (1.111 to 1.255)
Glycine to phosphate ratio	18	Weighted median	**<0.001**	1.197 (1.119 to 1.280)
	18	Inverse variance weighted	**<0.001**	1.147 (1.072 to 1.228)
	18	Weighted mode	**<0.001**	1.198 (1.118 to 1.284)
Adenosine 5'-monophosphate to glycine ratio	8	Weighted median	**<0.001**	0.743 (0.661 to 0.834)
	8	Inverse variance weighted	**<0.001**	0.777 (0.705 to 0.857)
	8	Weighted mode	**0.002**	0.731 (0.641 to 0.833)
Adenosine 5'-monophosphate to serine ratio	4	Weighted median	**0.005**	0.642 (0.472 to 0.873)
	4	Inverse variance weighted	**<0.001**	0.553 (0.412 to 0.742)
	4	Weighted mode	0.153	0.664 (0.435 to 1.012)

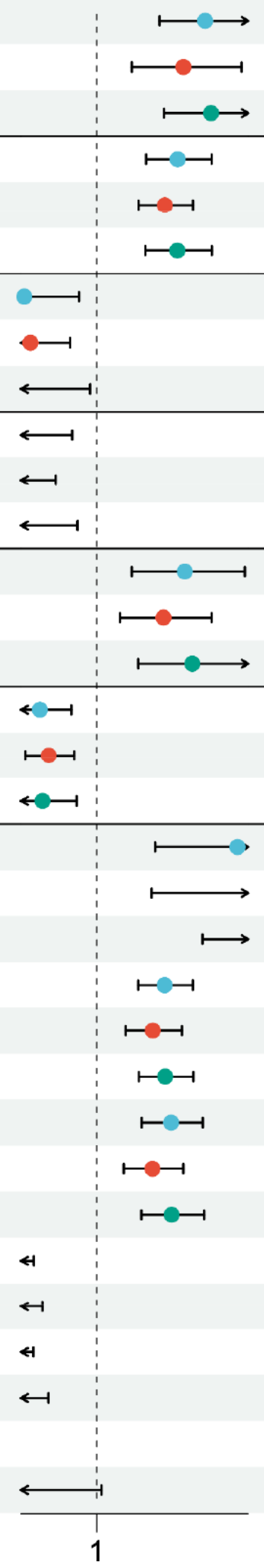

Figure S13. Forest plot showing additional significant positive Mendelian randomization associations among the 1,458 metabolites evaluated in this study.

Supplementary table 1. Baseline characters of 48 patients who provided anonymous clinical data from the Nephroseq database.

Variables	Discovery set<br>N=53
**Age (y)**	48.3 ± 18.7
**Gender (male/female, n)**	24 / 24
**Serum creatinine (mg/dl)**	0.96 ± 0.41
**Blood urea nitrogen (mg/dl)**	16.7 ± 8.4
**Primary disease (n)**	
**IgA nephropathy**	15
**Membranous nephropathy**	7
**Minimal change nephrotic syndrome**	4
**Membranoproliferative glomerulonephropathy**	3
**Diabetic nephropathy**	2
**Lupus nephritis**	6
**Amyloidosis**	3
**ANCA-related glomerulonephropathy**	2
**Others**	6
**Histopathology (Grade 0–4)**	
**Tubular cell damage grade**	1.9 ± 1.1 (1–4)
**Tubulointerstitial fibrosis grade**	2.1 ± 1.0 (1-4)

Supplementary table 2. Baseline characters of 36 patients with kidney biopsies collected during routine care at Xijing hospital from 2020 to 2023.

**Variables**	**IgAN group**	**Control group**
**Age (y)**	33.07±6.76	55.78±13.37
**Gender (male/female, n)**	16/7	5/8
**Serum creatinine (mg/dl)**	216.41±164.41	74.89±21.07
**Blood urea nitrogen (mg/dl)**	10.81±7.51	5.18±1.47
**eGFR [mL/(min·1.73m^2)]**	46.86±30.09	80.83±30.92
**Proteinuria (mg/24h)**	2682.11±2553.83	NA
**Albumin (g/L)**	36.03±7.06	44.74±3.82
**Uric acid (mmol/L)**	424.15±102.45	312.89±59.75
**Hemoglobin (g/L)**	130.22±27.56	136.13±16.32
**BMI (Kg/m^2)**	24.79±4.02	23.09±1.07

Supplementary table 3. Antibodies utilized in this study

**Reagent**	Company	Accession numbers
**β-actin**	Proteintech	81115-1-RR
**LDHA**	Proteintech	66287-1-Ig
**HK2**	Proteintech	22029-1-AP
**α-SMA**	Servicebio	GB13044-50
**Vimentin**	Proteintech	60330-1-Ig
**Twist1**	Proteintech	25465-1-AP
**ZO-1**	Proteintech	21773-1-AP
**PKM2**	Abclonal	A20991
**H3K18lac**	PTM BIO	PTM-1427RM
**H3K14lac**	PTM BIO	PTM-1414RM
**H4K12lac**	PTM BIO	PTM-1411RM
**Pan-lac**	PTM BIO	PTM-1401RM
**H4K5lac**	PTM BIO	PTM-1407RM
**H3K56lac**	PTM BIO	PTM-1421RM
**H4K8lac**	PTM BIO	PTM-1415RM
**Secondary Antibody (anti-mouse)**	Abclonal	AS003
**Secondary Antibody (anti-rabbit)**	Abclonal	AS014
**Pan- Acetyllysine**	PTM BIO	PTM-105RM

Supplementary table 4 Primer sequences utilized in this study.

**Gene**	**PCR primers information**	**Sequence (5' -> 3')**
Hk2	*Hk2* mouse forward primer	ATCTACGCCATTCCCGAGGACA
Hk2	*Hk2* mouse reverse primer	ATCTCTGCCTTCCACGCCACT
HK2	*HK2* human forward primer	GAGCCACCACTCACCCTACT
HK2	*HK2* human reverse primer	CCAGGCATTCGGCAATGTG
Ldha	*Ldha* mouse forward primer	ATGGCAACCCTCAAGGACCAG
Ldha	*Ldha* mouse reverse primer	CATGCCAACAGCACCAACCC
LDHA	*LDHA* human forward primer	TTGACCTACGTGGCTTGGAAG
LDHA	*LDHA* human reverse primer	GGTAACGGAATCGGGCTGAAT
Atca2	*Atca2* mouse forward primer	CTGACAGAGGCACCACTGAA
Atca2	*Atca2* mouse reverse primer	CATCTCCAGAGTCCAGCACA
Actb	*Actb* mouse forward primer	GGCTGTATTCCCCTCCATCG
Actb	*Actb* mouse reverse primer	CCAGTTGGTAACAATGCCATGT
ACTB	*ACTB* human forward primer	GAGCTACGAGCTGCCTGACG
ACTB	*ACTB* human reverse primer	CCTAGAAGCATTTGCGGTGG
Vim	*Vimentin* mouse forward primer	AGGTGGATCAGCTCACCAATGACA
Vim	*Vimentin* mouse reverse primer	TCAAGGTCAAGACGTGCCAGAGAA
TWIST1	*Twist1* human forward primer	GTCCGCAGTCTTACGAGGAG
TWIST1	*Twist1* human reverse primer	GCTTGAGGGTCTGAATCTTGCT
